# Heart Rate Variability and Cardiac Vagal Tone in Psychophysiological Research – Recommendations for Experiment Planning, Data Analysis, and Data Reporting

**DOI:** 10.3389/fpsyg.2017.00213

**Published:** 2017-02-20

**Authors:** Sylvain Laborde, Emma Mosley, Julian F. Thayer

**Affiliations:** ^1^Institute of Psychology, Department of Performance Psychology, German Sport University CologneCologne, Germany; ^2^Normandie Université Caen, UFR STAPS, EA 4260Caen, France; ^3^Southampton Solent UniversitySouthampton, UK; ^4^Bournemouth UniversityBournemouth, UK; ^5^Ohio State UniversityColumbus, OH, USA

**Keywords:** heart rate variability, parasympathetic nervous system, vagal activity, vagal tone, cardiac vagal control, parasympathetic activity

## Abstract

Psychophysiological research integrating heart rate variability (HRV) has increased during the last two decades, particularly given the fact that HRV is able to index cardiac vagal tone. Cardiac vagal tone, which represents the contribution of the parasympathetic nervous system to cardiac regulation, is acknowledged to be linked with many phenomena relevant for psychophysiological research, including self-regulation at the cognitive, emotional, social, and health levels. The ease of HRV collection and measurement coupled with the fact it is relatively affordable, non-invasive and pain free makes it widely accessible to many researchers. This ease of access should not obscure the difficulty of interpretation of HRV findings that can be easily misconstrued, however, this can be controlled to some extent through correct methodological processes. Standards of measurement were developed two decades ago by a Task Force within HRV research, and recent reviews updated several aspects of the Task Force paper. However, many methodological aspects related to HRV in psychophysiological research have to be considered if one aims to be able to draw sound conclusions, which makes it difficult to interpret findings and to compare results across laboratories. Those methodological issues have mainly been discussed in separate outlets, making difficult to get a grasp on them, and thus this paper aims to address this issue. It will help to provide psychophysiological researchers with recommendations and practical advice concerning experimental designs, data analysis, and data reporting. This will ensure that researchers starting a project with HRV and cardiac vagal tone are well informed regarding methodological considerations in order for their findings to contribute to knowledge advancement in their field.

## Introduction

**FIGURE 1:**
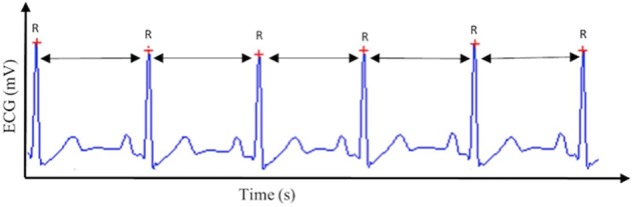
**Heart rate variability (HRV)**. This figure displays the way HRV is calculated based on the R–R intervals of the QRS complex extracted from the electrocardiogram (ECG) signal.

Thanks to more accessible technology (hardware and software) and since the establishment of standards by the Task Force of the European Society of Cardiology and the North American Society of Pacing and Electrophysiology two decades ago (Malik, 1996), followed by concurring guidelines from the Society for Psychophysiological Research (Berntson et al., 1997), heart rate variability (HRV), representing the change in the time interval between successive heartbeats (see **Figure 1**), became a strong focus of psychophysiological research. This is due to the fact that HRV provides an index of the parasympathetic nervous system (Malik, 1996; Chapleau and Sabharwal, 2011). This is of particular interest, given the association of the parasympathetic nervous system with many aspects relevant for psychophysiology, such as self-regulation mechanisms linked to cognitive, affective, social, and health phenomena (Porges, 2007; Thayer et al., 2009; McCraty and Shaffer, 2015). The vagus nerve is the main nerve of the parasympathetic nervous system (Brodal, 2010), therefore we refer to parasympathetic activity as vagal tone from now on. More specifically, in this paper we refer to cardiac vagal tone as assessed by HRV measurement (also referred to as cardiac vagal control, given it reflects the contribution of the vagus nerve to cardiac functioning). Even if we refer in this paper to broad recommendations on HRV, where possible we will specify when our recommendations apply specifically to vagal tone, given its relevance for psychophysiology.

Progressions in technology and computer science have made data collection and analysis of HRV very accessible to psychophysiology researchers interested in the phenomena. Moreover, HRV represents a non-invasive, pain free, economic and simple measurement which again attracts many researchers. However, this ease of access to HRV collection often obscures the complicated nature of understanding and correctly interpreting the huge range of information that is provided by the numerous HRV parameters. Therefore the very nature of HRV itself may have led to some confusion for its use in psychophysiological research so far. HRV measurement is very sensitive to several methodological aspects, which hinders the comparison between studies. Therefore, there is a need for researchers to be aware of methodological issues related to HRV measurement, so results can be compared across laboratories around the world. The aim of this paper is to provide an overview of the methodological aspects to consider for the use of HRV in psychophysiological research, with specific recommendations for experiment planning, data analysis, and data reporting. Before going further, we clearly state that our recommendations are not to become recognized standards or to enforce experimental procedures to assess HRV and cardiac vagal tone, because the answers will ultimately depend on the research questions. Instead, we aim to take the researcher by the hand and provide a comprehensive overview of the important methodological issues one has to consider for psychophysiological research on vagal tone. Therefore we provide the rationale for practical recommendations for the reader to make educated choices on those aspects. Given this overarching aim, we will not extensively cover every single topic in depth, instead we provide the reader with Supplementary Materials in order to visualize suitable resources and signpost to other relevant research.

When a budding researcher first searches for research conducted in HRV they are faced with a rather extensive list. On the 11th of January 2017, a search on the Web of Science with the keywords “HRV” returned 21,621 results, “parasympathetic” returned 10,983 results, “vagal” returned 16,339 results, while a combination of the three keywords returned 40,731 unique results. Therefore a psychophysiology researcher who wants to start investigating HRV and vagal tone is thus faced with a huge puzzle and may start to consult previous summative work (Malik, 1996; Berntson et al., 1997; Chambers and Allen, 2007; Quintana and Heathers, 2014; Shaffer et al., 2014; Billman et al., 2015; Sassi et al., 2015; Quintana, 2016; Quintana et al., 2016).

As mentioned earlier, measurement and interpretation issues were discussed two decades ago by a Task Force on HRV (Malik, 1996) and by further researchers (Berntson et al., 1997), offering the first solid foundation for HRV research. The recommendations of the Task Force are very useful and provide information on which HRV parameters to take into account and what their significance is at the physiological level. However, researchers in psychophysiology could not see a direct interpretation regarding the phenomena they were used to working with. Fortunately, some pioneers contributed then to the establishment of links between HRV and psychophysiological phenomena (Grossman and Taylor, 2007; Porges, 2007; Thayer et al., 2009; McCraty and Childre, 2010; Lehrer, 2013), giving birth to five psychophysiological theories that we detail in the next section. At the methodological level, the Task Force left researchers in psychophysiology to make their own decisions concerning experiment planning and the practical realization of experiments. In addition the data analysis and data reporting was also omitted of the Task Force report, which can both have a key influence on the results being reported and their interpretation.

A decade after the Task Force, an important milestone for research in psychophysiology was published. A special issue on cardiac vagal control addressed theoretical and methodological issues directly relating to psychophysiology (Chambers and Allen, 2007). Finally, an updated vision on HRV, including the theoretical and methodological progressions of the last two decades, was delivered by several research teams. A critical review of the new HRV analyses methods that emerged since the Task Force regarding HRV was provided by Sassi et al. (2015). Further, methodological issues were debated in a special issue lead by Tak et al. (2009) and Billman et al. (2015), interpretation and theoretical issues were presented in Shaffer et al. (2014), guidelines for reporting HRV experiments were presented in Quintana et al. (2016), statistical considerations for HRV case-control studies were presented in Quintana (2016), and methodological considerations for biobehavioral research were presented in Quintana and Heathers (2014). These reviews were helpful as they updated the knowledge acquired from two decades regarding HRV, however, they still did not fully answer the needs of psychophysiological researchers. The review of Sassi et al. (2015) concluded that the new methods to analyze HRV did not allow for better understanding of the physiological systems underlying HRV such as vagal tone, and therefore did not represent a meaningful added value for psychophysiologists. Billman et al. (2015) focused on methodological concerns mainly linked to research in physiology, which sometimes do not entirely portray the needs of psychophysiology researchers. Tak et al. (2009) focused on the methodological quality of HRV studies within a specific topic (i.e., functional somatic disorders). Shaffer et al. (2014) did focus on psychophysiological aspects reviewing the psychophysiological theories, but did not address issues related to measurement and data analysis. Quintana et al. (2016) provided a useful checklist for reporting articles on HRV for psychiatry, however, this very focus on data reporting in psychiatry left many questions for psychophysiological researchers. One of the key differences between the needs of psychiatry researchers in comparison to psychophysiological researchers is the focus on cases vs. control for the formers, and the focus on tasks vs. baseline for the latter ones. This focus on cases vs. control participants was also found in Quintana (2016), regarding the statistical considerations about reporting and planning HRV studies. Finally Quintana and Heathers (2014) provided very helpful methodological recommendations for psychophysiological research regarding within-subject design, controlling for respiration and baseline measurement. However, they did not account for many other additional issues that need to be addressed by a psychophysiological researcher in order to plan, measure, analyze, interpret and report HRV data correctly. Several of these aspects were later attended to by Quintana et al. (2016), however, there is still a need to provide a comprehensive overview. This overview should cover the majority of methodological challenges faced by a psychophysiological researcher willing to investigate HRV and vagal tone, a need which this paper aims to fill.

In summary, the initial ease of access of HRV often hides the complicated nature of HRV in order to understand and correctly interpret which information is actually relevant. HRV measurement is very sensitive to several methodological aspects, such as the body position baseline is taken from (Young and Leicht, 2011), which hinders the comparison between studies. Moreover, the inconsistent reporting of HRV parameters and analyses in HRV research papers may contribute to the confusion in the use of HRV and the conclusions drawn regarding vagal tone in psychophysiological research so far, as pointed out by Quintana et al. (2016). Thus the aim of this paper is to provide recommendations for the assessment of HRV in psychophysiological research. It will endeavor to cover all aspects of HRV research in psychophysiology from experiment planning, measurement, data analysis and data reporting. In other words it will act as a set of recommendations in order to enable psychophysiological researchers to conduct a full research project with HRV with a focus on vagal tone.

## HRV in Psychophysiological Research: A Focus on Vagal Tone

As reviewed by Shaffer et al. (2014), there are five theories implying HRV in psychophysiological research: the neurovisceral integration model (Thayer et al., 2009), the polyvagal theory (Porges, 2007), the biological behavioral model (Grossman and Taylor, 2007), the resonance frequency model (Lehrer, 2013), and the psychophysiological coherence model (McCraty and Childre, 2010). The neurovisceral integration model assumes a connection between the prefrontal cortex and the heart through the central autonomic network and the vagus nerve (Thayer et al., 2009). The main assumption of this model is that the higher the vagal tone, the better executive cognitive performance, as well as better emotional and health regulation (Thayer et al., 2009). Porges (2007), who developed the polyvagal theory, assumed that a higher vagal tone is associated to better social functioning. The biological behavioral model (Grossman and Taylor, 2007) focuses on the fact that vagal tone plays a primary role in regulation of energy exchange by synchronizing respiratory and cardiovascular processes during metabolic and behavioral changes. A higher resting vagal tone is seen as adaptive, given “it reflects a functional energy reserve capacity from which the organism can draw during more active states” (Grossman and Taylor, 2007, p. 279). Lehrer (2013) put forward the resonance frequency breathing model which mentions that an efficient way to increase vagal tone is through slow paced breathing at the resonance frequency. Finally, the psychophysiological coherence model (McCraty and Childre, 2010) shares similarities to Lehrer, in that a higher vagal tone can be achieved through slow paced breathing. They also postulate that slow paced breathing coupled with positive emotions will produce a broad range of positive outputs linked to personal, social, and global health (McCraty and Childre, 2010). One common ground of those five theories regarding HRV is their focus on vagal tone, which also constitutes one of the main focuses of HRV research. Given the focus on vagal tone of all existing psychophysiological theories, its measurement and interpretation will receive particular attention within this paper.

## Planning a Research Project with HRV

In this first section on experiment planning we will address the issues concerning the HRV variables to assess, the choice of within-subject vs. between-subject design, sample size, experiment structure, variables to control and the choice regarding considering HRV as a dependent or independent variable.

### HRV Variables to Assess

The first question that the researcher wants to answer is the following: out of the more than 70 variables that can be calculated from HRV analysis (Bravi et al., 2011; Smith et al., 2013a,b) – what are the variables of interest for psychophysiological research? The answer will depend of the phenomenon of interest and the research question itself. As an overview, HRV analysis can be performed in the time-domain, in the frequency-domain and finally with non-linear indices. We will present the main variables of interest for psychophysiological researchers with the physiological systems that they reflect (**Table 1**), with a specific interest on variables depicting vagal tone given the theoretical focus existing on those variables.

**Table 1:** Summary of the main heart rate variability parameters and their physiological origin.

	Variable	Description	Physiological origin
Time-domain	SDNN	Standard deviation of all R–R intervals	Cyclic components responsible for heart rate variability
	RMSSD	Root mean square of successive differences	Vagal tone
	pNN50	Percentage of successive normal sinus RR intervals more than 50 ms	Vagal tone
	Peak-valley	Time-domain filter dynamically centered at the exact ongoing respiratory frequency	Vagal tone
Frequency-domain	ULF	Ultra-low frequencies	Circadian oscillations, core body temperature, metabolism and the renin-angiotensin system
	VLF	Very-low frequencies	Long-term regulation mechanisms, thermoregulation and hormonal mechanisms
	LF	Low frequencies	Mix of sympathetic and vagal activity, baroreflex activity
	HF	High frequencies	Vagal tone
	LF/HF	Low frequencies/high-frequencies ratio	Mix of sympathetic and vagal activity
Non-linear indices	SD1	Standard deviation – Poincaré plot Crosswise	Unclear, depicts quick and high frequent changes in heart rate variability
	SD2	Standard deviation – Poincaré plot Lengthwise	Unclear, depicts long-term changes in heart rate variability

In the time-domain, the standard deviation of all R–R intervals (SDNN) reflects all the cyclic components responsible for variability in the period of recording (Malik, 1996). The root mean square of successive differences (RMSSD) reflects vagal tone (Thayer and Lane, 2000; Kleiger et al., 2005) and is highly correlated with high-frequency (HF) HRV (Kleiger et al., 2005). Finally, RMSSD is relatively free of respiratory influences, contrary to high frequency parameters (Hill and Siebenbrock, 2009). The percentage of successive normal sinus RR intervals more than 50 ms (pNN50) is correlated with RMSSD and HF power and thus supposed to reflect also vagal tone (Shaffer et al., 2014). However, the RMSSD typically provides a better assessment of vagal tone and it is normally preferred to pNN50 (Otzenberger et al., 1998). Beyond those traditional variables, additional analyses based on the time-domain properties of the ECG signal can be used to infer vagal tone, such as the peak-valley analysis (Grossman et al., 1990), also known as peak-to-trough analysis (Lewis et al., 2012), which acts as a time-domain filter dynamically centered at the exact ongoing respiratory frequency (Grossman et al., 1990). Finally, another way of quantifying vagal tone is by using the Porges-Bohrer method (Lewis et al., 2012), which displayed interesting statistical properties in comparison to other metrics.

In the frequency-domain, the analysis requires filtering the signal into different bands (for a comprehensive visual display of the filtered frequencies, see Shaffer et al., 2014, p. 8). The ultra-low frequencies (ULF) band is located below 0.0033 Hz. It reflects circadian oscillations, core body temperature, metabolism and the renin-angiotensin system (Berntson et al., 1997). It can be only assessed with 24 h recordings (Kleiger et al., 2005). The very-low frequency (VLF) range is located between 0.0033 and 0.04 Hz. This band represents long-term regulation mechanisms, thermoregulation and hormonal mechanisms (Malik, 1996; Berntson et al., 1997). The low-frequency (LF) band ranges between 0.04 and 0.15 Hz. The LF band reflects a mix between sympathetic and vagal influences that shows an influence of both sympathetic and parasympathetic branches (Malik, 1996; Berntson et al., 1997). HF, specifically between 0.15 and 0.40 Hz (Malik, 1996), reflects vagal tone. This is also frequently called the respiratory band because it corresponds to the heart rate variations related to the respiratory cycle (Eckberg and Eckberg, 1982). HF is influenced by breathing when breathing rates are between nine cycles per minute (0.15 Hz) and up to 24 cycles per minute (0.40 Hz) (Malik, 1996). When breathing remains between these cycles per minute then HRV stays between the boundaries of those frequencies, thus reflecting vagal tone. Bands might need to be adjusted regarding the population of interest: for example children and infants breathe faster, and a recommendation for them would be to move the boundaries of the band to 0.24–1.04 Hz at rest (Quintana et al., 2016). In a similar vein, athletes usually present slower respiratory rates that may interfere with of the measured HF band (Saboul et al., 2014). Therefore population characteristics should always be considered when bands are chosen, either by looking at previous research, or by calculating respiratory rates of the sample under investigation (Quintana et al., 2016). In this case it is always important to couple the HRV frequency analysis with other time-domain parameters supposed to index vagal tone to see to which extent they correlate, for example with RMSSD that is supposed to be less affected by respiratory influences (Hill and Siebenbrock, 2009).

Heart rate accelerates during inspiration and slows down during expiration, a phenomenon that is called respiratory sinus arrhythmia. Hence, in the literature the term respiratory sinus arrhythmia is often written instead of HF, as it is supposed to reflect vagal tone (Eckberg, 1983). However, we would recommend for clarity matters to refer to HF when referring to vagal tone, and use RSA to depict the heart rate variations accompanying inspiration and expiration, respectively, accelerating and slowing down (Eckberg and Eckberg, 1982). Finally, the LF/HF ratio was long considered as representing the sympatho-vagal balance which is the balance between the sympathetic and parasympathetic systems. However, this view has been highly criticized (Eckberg, 1997; Billman, 2013). Among the most critical aspects is the loose relationship between LF power and sympathetic nerve activation, and the non-linear and non-reciprocal relationship between sympathetic and parasympathetic nerve activity (Billman, 2013). Hence, there is now a consensus to say that the precise physiological underpinning is unclear, thus lowering its predictive value. Although around 65% of HRV papers are still basing their conclusions on it (Heathers, 2014), we strongly recommend researchers to adopt HRV indices that reflect clearly identified physiological systems with a theoretical underpinning such as the indices of vagal tone (i.e., RMSSD, peak-valley, and HF-HRV).

In addition, we could mention some non-linear indices that can be obtained from the interbeat interval (IBI) interval. As the autonomic nervous system is characterized by complex and erratic fluctuations, some researchers suggest that non-linear analyses might be more adequate and precise for HRV analysis than the prevalent linear measures (Piskorski and Guzik, 2005). One of those linear indices is the Poincaré plot. The plot itself displays the correlation of R–R intervals (which are usually measured in milliseconds) by assigning each following interval to the, respectively, former interval as a function value (auto-correlation). The result is a plot which illustrates quantitative and qualitative patterns of one’s individual HRV in the shape of an ellipse. Additionally, two other parameters are added to the ellipse, namely the two different standard deviations resulting from the orthogonal distances between the scatter and the elliptical diameters. Firstly crosswise (SD1) and secondly lengthwise (SD2) to the ellipse. SD1 is supposed to be more sensitive to quick and high frequent changes whereas SD2 is viewed as an indicator of long-term changes (Piskorski and Guzik, 2005). The result is a plot that illustrates quantitative and qualitative patterns of one’s individual HRV in the shape of an ellipse. As research results suggest, Poincaré plots can be seen as indicators of vagal activity and reduced cardiac vagal control which are associated not only with physiological but also with psychological strain and stress (Collins and Karasek, 2010; Melillo et al., 2011). However, some caution is still required regarding non-linear indices and their utility has still to be demonstrated to predict psychophysiological phenomena (Sassi et al., 2015). Hence, this would allude to not using them as single indicators, but rather as complementary HRV indicators.

Finally, a critical review of the new HRV analyses methods that emerged since the Task Force regarding HRV (Malik, 1996) was realized by Sassi et al. (2015), and the conclusion was that the new methods did not bring any additional information regarding physiological underpinnings of HRV, and therefore no additional information for vagal tone.

### Within-Subject Design vs. Between-Subject Design

Deciding the experimental design is crucial for HRV experiments. Given high inter-individual variations and the complex interactions influencing HRV, within-subject designs are highly recommended (Quintana and Heathers, 2014). Within-subject designs offer optimal experimental control, contribute to the elimination of individual differences in respiratory rates (though there is still a need to control for them, which will be covered later), require less participants given they offer an increased statistical power, and reduce the impact of external factors such as medication, alcohol, smoking, etc. (Quintana and Heathers, 2014). In case testing occurs on different days, the time when the experiment is realized should be maintained constant and this should also apply to between-subject designs, having participants take part to the experiment at the same time of the day (Massin et al., 2000; van Eekelen et al., 2004). One limitation of within-subject designs is the habituation to the experimental conditions and the learning effect that can be observed in some experimental tasks. Therefore in this case we would recommend whenever possible the use of non-identical correlated tasks investigating similar constructs. For example measuring response inhibition which can be investigated through the use of concurrent tasks such as the Stroop color naming task and a stop signal task (Miyake et al., 2000); or if the task needs to remain the same and the conditions change, it is then possible to counterbalancing the conditions in order to reduce confounding effects (e.g., low pressure vs. high pressure, such as in Laborde et al., 2014).

### Sample Size

An effect size distribution analysis of close to 300 HRV effect sizes (Quintana, 2016) revealed that HRV studies are generally underpowered, and that Cohen’s guidelines (Cohen, 1988) to interpret effect sizes should be adapted. More particularly, instead of interpreting 0.20, 0.50, and 0.80 as, respectively, small, medium, and large effect sizes, we should rather interpret 0.25, 0.50, and 0.90 as representing, respectively, small, medium, and large sizes. In terms of sample sizes, the effect size distribution analysis suggests that in order to achieve 80% power, samples of 233, 61, and 21 participants are required, respectively, to detect small, medium and large effect sizes (Quintana, 2016). If another effect size or statistical power level is desired, power analysis can be conducted for example with the help of the G^∗^Power 3 (Faul et al., 2007, 2009) or the “pwr” package (Champely, 2016) available for the R statistical package, which facilitates the calculation of required sample size.

### Experiment Structure and the Three Rs: Resting, Reactivity, Recovery

Following the previous section, the next question is to consider the structure of the experiment. But first of all, we need to understand the concepts of tonic or phasic HRV which are recognized to be important in terms of adaptation (Porges, 2007; Thayer et al., 2012). Tonic HRV has also been referred to as resting HRV or baseline HRV and is when HRV is taken at one time point. Phasic HRV shows how the system reacts and has been named reactivity, stimulus-response, change delta HRV and vagal withdrawal in order to represent change in HRV from two different time points. When assessing HRV in these two concepts, tonic and phasic, it is important to consider what the level or changes in HRV represent. For example when measuring tonic HRV it is clear from the literature that a higher resting vagal tone is beneficial in most of the cases (Thayer et al., 2012). There are some exceptions to this general rule (e.g., Stein et al., 2005; Peschel et al., 2016), for example when a higher resting vagal tone level is observed in the case of eating disorders such as bulimia nervosa (Peschel et al., 2016), which may be due to decreased resting metabolic rate originating from limited calorie intake (Martin et al., 2007). Assessing the phasic level may require a little more interpretation of the vagal activity in order to determine whether it is adaptive or not. For example a high level of vagal withdrawal (decrease in HRV) may be seen as adaptive or not depending on the situation. This may been seen as adaptive when the individual is facing a physical stressor or a mental stressor that does not involve executive function, as this demonstrates the individual’s ability to provide the organism with the necessary energy to face the stressor (Porges, 2007), as showed experimentally (Neumann et al., 2004; Rottenberg et al., 2005; Lewis et al., 2007; Messerotti Benvenuti et al., 2015). However, when the stressor faced by the individual requires executive functioning, then a higher level of vagal withdrawal is seen as maladaptive (Thayer et al., 2012), as showed experimentally (e.g., Marcovitch et al., 2010; Laborde et al., 2014, 2015b; Park et al., 2014). Related to this, an interesting recent study by Park et al. (2014) showed that tonic HRV might influence phasic HRV. In a selective attention task involving fearful and neutral faces to act as distractors, when using fearful distractors lower tonic vagal tone was associated with phasic vagal tone withdrawal, under both low and high perceptive load. While in contrast higher tonic vagal tone was associated with phasic vagal tone enhancement under low perceptual load and an absence of phasic HRV suppression under high perceptual load. As a consequence for researchers, this means that both tonic and phasic HRV values need to be taken into account because their interaction can shed light on findings that would otherwise remain unclear.

**FIGURE 2:**
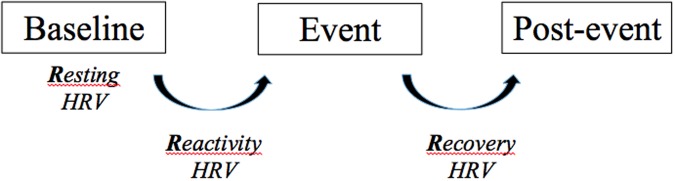
**Typical experiment structure for HRV experiments, depicting the three Rs: resting, reactivity, and recovery**. HRV, Heart rate variability.

Based on the respective role of tonic and phasic vagal tone, we would advise researchers to have the following structure in their experimental designs: three time points referred to as: baseline, event, and post-event (e.g., Berna et al., 2014). We suggest this experimental structure to subsequently introduce the three Rs of HRV: resting, reactivity, recovery (see **Figure 2**). By using the three Rs structure it allows for investigation of tonic HRV for each of the three measurement points (i.e., baseline, event, post-event). In addition it allows for a measure of phasic HRV as we can measure the change between baseline and event (that we coin here as “reactivity”), the change between task and post-event (that we coin here as “recovery”) and the change between baseline and post-event according to the research questions. The change in HRV, for reactivity and recovery, can be either reported in absolute values, or in percentage (see Duschek et al., 2009).

### Variables to Assess

Now the structure of HRV experiments has been finalized, it is important to consider any confounding variables influencing HRV that can be controlled. In the Supplementary Materials (Data Sheet 1) we provide the reader with an example of a demographic form that can be adapted and used within experiments. The content of which recommends collecting the following information and potentially controlling for them, according to the research question. Therefore we suggest the researcher considers the following stable and transient participant’s variables.

Stable variables:

• Age and gender (Umetani et al., 1998)
• Smoking (Hayano et al., 1990; Sjoberg and Saint, 2011)
• Habitual levels of alcohol consumption (Quintana et al., 2013a,b)
• Weight, height and waist-to-hip ratio (Yi et al., 2013)
• Cardioactive medication, such as antidepressant (Kemp et al., 2010), antipsychotic (Cohen et al., 2001) or antihypertensive (Schroeder et al., 2003). Among psychotropic medication, a systematic review revealed that only tricyclic antidepressant and clozapine were found to statistically significantly influence HRV (Alvares et al., 2016), but we would still recommend to document any cardio-related medication taken by the participants.
• Oral contraceptive intake for female participants, it might not influence HRV during rest conditions (Rebelo et al., 2011; Nisenbaum et al., 2014), but it may influence the response to stressful conditions (Kirschbaum et al., 1999).

Transient variables:

• Follow a normal sleep routine the day before the experiment, record the typical bed time and typical waking time (Stein and Pu, 2012)
• No intense physical training the day before the experiment (Stanley et al., 2013a)
• No meal the last 2 h before the experiment (Lu et al., 1999)
• No coffee – or caffeinated drinks such as energizing drinks – (Zimmermann-Viehoff et al., 2015) or tea (Inoue et al., 2003) in the 2 h before the experiment
• Ask if they need to use the bathroom before the experiment begins (Heathers, 2014)
• No alcohol for 24 h prior to the experiment (Quintana et al., 2013a,b)

Keeping track of all the potential confounding variables may allow the researcher to exclude participants prior to data collection or to understand outliers within the data post collection. As a note, individuals may give inaccurate information when reporting many of these demographic factors. Therefore researchers should bear in mind that the ideal procedure would be to get objective measures of these potentially confounding factors whenever possible. For instance, rather than asking participants if they have any blood pressure conditions, taking a direct measure of blood pressure would be more accurate. Also, instead of self-reporting physical/mental illnesses, it would be preferential for participants to receive a physical examination by a clinician and screening for psychiatric disorders.

### HRV as a Dependent or Independent Variable

Heart rate variability has been used as both a dependent or independent variable by researchers. As a dependent variable, it is used to see how it relates in correlations and regression analyses (e.g., Laborde et al., 2011; Jennings et al., 2015) or how it differentiates groups split according to other criteria like individual differences (e.g., Schwerdtfeger and Derakshan, 2010; Laborde et al., 2015a) or to experimental conditions (e.g., Egizio et al., 2008; Laborde and Raab, 2013).

As an independent variable, much research has considered resting HRV as an individual difference *per se*, and groups were then created with median split, for example low and high RMSSD (for a review, see Thayer et al., 2009). The rational for considering HRV as an individual difference can be seen as more than a statistical trick, because it has also some theoretical and empirical premises. For example high resting HRV, which often reflects high resting vagal tone, is consistently associated to positive outcomes (Thayer et al., 2009). Furthermore resting levels of HRV and particularly of vagal tone are reasonably stable in time (Bertsch et al., 2012) and finally cardiac vagal control is partially heritable (Neijts et al., 2014).

## Measurement Standards

In this category we address the measurement standards associated with best practice within HRV research. These include: issues associated with the HRV recording devices and the signal to record, electrocardiogram (ECG) sampling, duration of recording, baseline recording, measurement in ambulatory settings, measurement with movement, and respiration.

### HRV Recording Device and Signal

Several recording techniques exist to measure HRV either through ECG recordings, the IBI or photoplethysmography. Using an ECG allows researchers to directly obtain HRV data from the electrical stimulus of the heart that is seen as the QRS complex (the graphical depiction of ventricle depolarization, i.e., a heartbeat). This can be collected through more traditional ECG equipment or through modern technologies such as the eMotion Faros device (Mega Electronics, Kuopio, Finland) using only two electrodes. ECG recordings are more accurate in terms of artifact correction because they allow the researcher to physically see the QRS complex and hence the heart beats, leading to very precise correction. As we will detail later artifact correction is very important step in the pre-processing of the signal (Berntson and Stowell, 1998; Shaffer and Combatalade, 2013). In case electrodes are used, it is necessary to follow standard recommendations regarding electrodes positioning according to the device used (Kligfield et al., 2007), and also consider skin preparation in order to improve signal quality such as cleaning or hair removal.

When measuring the IBI the researcher solely collects the time between heart beats. One method of collecting IBI data is through chest belts. The advantage of these belts coupled to heart rate monitors is that they are widely spread, and some specific heart rate monitors can record the IBI interval properly (Weippert et al., 2010). The disadvantage of chest belts is that they will create more artifacts than electrodes due to friction against the skin. In addition, they can only measure the IBI and not the ECG signal, making it less accurate than ECG.

Photoplethysmography involves shining a small light onto an area where capillaries are easy to access either through the finger or ear lobe using a sensor. Then the light reflected back to the sensor depicts blood volume in the vessel and thus forms the grounds of a heartbeat. Photoplethysmography measures pulse-to-pulse interval data, which is a mixture of the IBI and pulse transit time. Photoplethysmography is considered to represent an accurate approximation of the IBI (Gil et al., 2010). A comparative review of photoplethysmography against ECG stated that photoplethysmography can be used during rest but not during stress (Schafer and Vagedes, 2013). This is because stress induces changes in pulse transit time (the time the blood pressure wave takes to travel from the heart to the periphery) which results from changes in the elasticity of the arteries which cannot be detected through IBI (Shaffer et al., 2014). Furthermore, the curved peak of the blood volume pulse signal is harder to detect accurately than the sharp upward spike of the R wave of the QRS complex which can best be determined through ECG. Finally, the emergence of the quantified self movement (Swan, 2013) – lay people willing to track and monitor their own psychophysiological data – is giving birth to many consumer devices aiming to measure HRV. However, for the majority, the consumer devices have not been validated against ECG measures, and present several drawbacks (Quintana et al., 2016). These usually include: reporting a proprietary metric rather than a standard metric, not providing access to raw data, and not offering technical details of correction methods (Quintana et al., 2016). Therefore, for research purposes we would recommend using electrodes instead of a chest belt or finger sensors. Researchers should aim to obtain the ECG recording to allow for precise editing of the signal for artifact correction. Importantly, in case the device used does not allow for the use of time markers, a very precise time protocol of every experimental event should be kept in order to allow for later analysis (see an example in Supplementary Material, Data Sheet 2).

At the applied level for practitioners, with the growing impulse to quantify psychology and with the growth of biofeedback, it is not surprising that many practitioners look to HRV smartphone apps to meet this need. The development of smartphone apps connected to either a chest belt or photoplethysmography to measure HRV, such as Ithlete, have been shown to provide reliable HRV measurement (Flatt and Esco, 2013). However, this was based on the RR interval and for research purposes we recommend the use of devices able to record the ECG signal, as mentioned earlier. We strongly recommend to avoid using smartphone apps based on the camera of the smartphone to detect the finger pulse because their sampling is simply too low to provide a reliable assessment of HRV. Considering sampling rates is crucial to ensure accurate ECG recording, as we will discuss in the next session.

### ECG Sampling

Together with the choice of HRV recording technique it is also important to make an informed choice of ECG sampling. Temporal accuracy is crucial to calculate successfully the variance of a time series (Quintana et al., 2016), to identify as precisely as possible the fiducial points (i.e., landmarks) of the ECG complex. The accuracy of HRV measurements is primarily determined by the sampling rate of the data acquisition system, which should be set at a minimum of 200 Hz according to the Task Force (Malik, 1996). More conservative guidelines advise between 500 and 1000 Hz (Riniolo and Porges, 1997; Berntson et al., 2007), which may be particularly relevant in case of lower amplitude RSA (Riniolo and Porges, 1997). However, recent development on this topic, relying on Monte Carlo-based analysis of false positive rates, showed that when R-peak interpolation (i.e., mathematical estimation of the digitized signal performed to enhance the R-wave fiducial point) was applied prior to HRV calculation, sampling rate could even be lower than 100 Hz, and without R-peak interpolation the analysis could be considered as reliable down to 125 Hz for all measures, and far lower in case of specific measures (Ellis et al., 2015). Based on these recent developments, we advise a ECG sampling rate of at least 125 Hz for researchers in psychophysiological research in order to ensure a reliable assessment of HRV parameters, but researchers who would like to anticipate issues that may arise with low amplitude RSA could choose as a conservative lower boundary of 500 Hz.

### Duration of Recording

As duration of recording might have an influence on HRV parameters, above all for the time-domain, the Task Force created a set of gold standards in terms of time measurement durations. They recommend for short-term recording the duration of 5 min (Malik, 1996) in order to ensure comparability of results across studies and laboratories. The basis for the recommendation is that the recording should last for at least 10 times the wavelength of the lower frequency bound of the investigated component. In specific cases, for example to meet the needs of an experimental design, recordings could be made shorter. However, the argumentation to do so needs to be strong and 1 min should be seen as the absolute minimum to obtain a reliable assessment of HF (Malik, 1996). More recently this 5 min gold standard has been challenged with shorter recording durations. For example a 1 min recording of the natural log of RMSSD has been proven to offer good reliability in comparison to the classical 5 min RMSSD (Esco and Flatt, 2014). Furthermore recordings between 10 and 50 s, according to which HRV parameters are considered, have been proven to be reliable under certain conditions (Salahuddin et al., 2007). Very recent work by Munoz et al. (2015) investigated the validity of ultra-short and short recordings on HRV measurements in a very large adult sample (*N* = 3,387). They found that it is unnecessary to use recordings longer than 120 s to obtain accurate measures of RMSSD. In addition, even a single 10 s (standard ECG) was found to yield a valid RMSSD measurement, although an average over multiple 10 s ECGs is preferable. Those 10 s periods don’t need to be contiguous (i.e., not in succession), so it is possible to obtain a good estimate from several 10 s measurements spread over a trial or the experiment. In any case researchers would need to carefully justify their choice of periods duration and location within the experiment when presenting their data analysis strategy. In summation from the suggestions presented for duration of recording, we would recommend in line with the Task Force (Malik, 1996) when possible to keep a 5 min recording to enable comparison between clinical studies. Depending on the research question a minimum duration of 1 min when vagal tone is targeted to allow frequency analysis and also according to the research question consider shorter recordings if RMSSD is used as an index of vagal tone. This would reduce the duration of HRV experiments and allow for ultra-short measurements in specific cases, for example enabling genetic epidemiological studies to be performed on a large scale.

For certain indicators, longer recordings of 24 h could be interesting. The Task Force mentions that the 24 h indices seem to be “stable and free of placebo effect, (which may make them) ideal variables with which to assess intervention therapies” (Malik, 1996, p. 363). However, a main difference has to be taken into consideration for the analysis and that is whether analyses should be based on a single segment of 24 h, or on a 5 min epochs over a 24 h period. Single analysis of 24 h suffer from several problems (Malik, 1996). Firstly they violate stationarity; if mechanisms responsible for heart period modulation of a certain frequency remain unchanged during the whole recording period, then – and only then – the corresponding frequency component of HRV may be used as a measure of these modulations, otherwise the interpretation can’t be ascertained. Secondly, they do not reflect the activity of the autonomic nervous system. For example, Roach et al. (1998, 2004) and Raj et al. (2004) have shown that low frequency HRV measures (e.g., ultra-low frequency and SDANN) reflect physical activity and functional capacity of patients and not strictly the autonomic nervous system activity. Therefore interpretations of differences between individuals on these measures as indicative of autonomic nervous system differences are problematic (Thayer et al., 2006). In the case of 5 min epochs over 24 h period, this can be very advantageous as we can examine circadian variation and night values which in some cases can be more predictive than daytime values (e.g., Jarczok et al., 2012). Those 5 min epochs can be used differently: either using a moving window, for example a 5 min moving window to calculate HRV parameters at 1 min intervals (e.g., Fenton-O’Creevy and Lins, 2012), or using strict intervals, for example 5.35 min blocks (Jarczok et al., 2012). We would advise to use strict intervals, given the moving average suffers from interpretive difficulties. Afterward, average on 24 h can be calculated, as well as day and night averages according to the research question (Jarczok et al., 2012). In previous 24 h monitoring bulky Holter devices were previously used which may cause some discomfort for participants (e.g., Soares-Miranda et al., 2014). However, the new generation of ECG devices weighting under 15 g (for example, the Faros devices from Mega Electronics, Kuopio, Finland) remain almost unnoticed by participants.

### Baseline Recording

The recording of an accurate baseline is crucial (Quintana and Heathers, 2014), for several reasons. In order to standardize baseline measurement it should be made as consistent as possible to ensure comparability of results across samples, experiments, and laboratories. The way in which it is realized should be through precisely described methods (e.g., body posture and instructions given). In the majority of studies, the baseline recording is generally taken while sitting with knees at a 90° angle, both feet flat on the floor, hands on thighs and eyes closed, similar to what is recommended for blood pressure procedures (Pickering et al., 2008; Ghuman et al., 2009). With regard to hand position we recommend palms facing upward, given palms facing downward could introduce interoceptive effects if participants feel their radial (wrist) pulse. Other postures for baseline [e.g., supine (lying down), standing] might be used if they make sense regarding the experimental conditions, for example in sleep research (Neijts et al., 2014). Whatever the posture chosen, it is important that before the baseline measurement, the participant has been in this posture at least for 5 min (Ghuman et al., 2009). This can be referred to more generally as acclimatization to the recording environment, which is accomplished by using an analysis period starting later than the start of the recording, which ensures that the potential anxiety and increased attention to respiration and heart rate that may occur when people are told that recording is starting already potentially fade out (Quintana et al., 2016). In addition, this would support not announcing the start of recording to protect the validity of the measurement period of interest. During baseline recording participants have to stay seated without speaking or making any movements, they are asked to relax and to breathe spontaneously. The best is to control for the time of day of the assessment (van Eekelen et al., 2004) and to make procedures consistent throughout the participants.

Some solutions were created to standardize baseline with the double aim to avoid mind-wandering, disruptive thoughts and to propose a baseline that is closer to experimental conditions. Firstly, some researchers attempted to standardize baseline recording using a video with neutral stimuli, like an aquatic video, which may be more comfortable for some participants than sitting with their eyes closed (Piferi et al., 2000). However, researchers have to be conscious that this can also influence individual’s cardiac reactivity to the experimental tasks. Secondly, a passive and restful baseline is often compared to experimental tasks that involve performance of a psychomotor, cognitive, or stressful task for example. This might then conflict the difference between passive rest in regards to paying attention to the experimental task, with the difference between passive rest in regards to the specific experimental task demands (Quintana and Heathers, 2014). An alternative to this forced relaxation could be then to perform the Vanilla baseline (Jennings et al., 1992), where participants have to perform a task requiring sustained attention but minimal cognitive load. However, researchers have to make this decision carefully in regards to their experimental task as sustained attention is still linked to HRV (Thayer et al., 2009).

To summarize, some researchers argue that an ideal HRV baseline recording does not exist, as there is not a correct list of parameters that would apply under all circumstances. Instead the following definition explains the parameters for a baseline recording, “the non-task situation that best controls for the presence of task comparison” (Quintana and Heathers, 2014, p. 6) and we agree that this is suitable where experiments are concerned. In the case that we want to consider resting HRV as predictor we still need to standardize the procedure, which is generally the one indicated earlier (i.e., sitting, knees at a 90° angle, hands on thighs, palms facing upward, eyes closed). According to the experimental condition, we recommend the body position to be as close as possible as to the one used during baseline. For example sitting baseline compared to a sitting cognitive task or a standing baseline compared to a standing psychomotor task. Finally, when baseline recording is used to assess HRV as a trait, the aggregation across at least two measurements is recommended in order to discard situation specific variance (Bertsch et al., 2012). Regarding this last point, some authors recommend to measuring HRV in supine resting first thing in the morning so it is the less influenced by external factors (Buchheit et al., 2005).

### HRV in Ambulatory Settings

We have previously mentioned that standardization for HRV experiments is crucial and therefore running reliable lab experiments is important in HRV research. However, researchers in psychophysiological research may consider the ecological environmental impacts on HRV and thus may ask themselves whether ambulatory, long-term measurement of HRV is possible. The answer is yes and it can provide very interesting information, for example when assessing 24 h recordings. When assessing HRV *in situ*, the predictive value of HRV is increased when controlling for respiration and physical activity (Wilhelm et al., 2004). Ambulatory settings will introduce the issue of dealing with movement, which we address in the next section.

### HRV Recording and Movement

In line with the previous section, we now consider whether it is possible to record HRV with movement, either inside or outside the lab. As HRV reflects the activity of the sympathetic and parasympathetic nervous systems, when the individual starts to move it affects immediately HRV, as both systems are involved in meeting physical demands (Brodal, 2010). Therefore movement will influence HRV parameters and in addition it may cloud the regulation linked to cognitive, emotional, social and health processes. The guidelines of the Task Force (Malik, 1996) are extremely clear on this matter: for an unambiguous interpretation of the physiological mechanisms underlying HRV, the measurement needs to be realized without physical activity. However, if the research question requires it, it may be possible to perform ambulatory measurements of HRV while controlling for respiration and physical activity (Grossman et al., 2004), however, researchers have to be aware that in this case a clear interpretation of vagal tone won’t be possible.

Another issue with movement is that we risk more artifacts within the data set. Currently, there is no generally available strategy to compute algorithms that are able to separate the influence of movement on HRV from the influence of other regulatory processes (but see Verkuil et al., 2016, for a new approach to this issue). The most common strategy in case of movement is to collect accelerometer data together with the HRV measurement and then to delete the sections where movement was excessive (e.g., Hansen et al., 2003; Johnsen et al., 2003). Specific algorithms like continuous wavelet transformation minimizes motion artifact (Villarejo et al., 2013), however, this only addresses one aspect of the issue because concerns related to interpretation of the data remain (Malik, 1996). As an alternative, HRV can be assessed directly before the task involving movement, for example when used as a precompetitive marker before sport competition where a decrease in vagal tone is generally observed, like before a swimming competition (Cervantes Blásquez et al., 2009) and a bike (i.e., BMX) competition (Mateo et al., 2012). HRV assessed before physical performance could then potentially serve to some extent as a predictor for the following motor performance. Building on this, some studies evidenced the predictive role of HRV measured during the task in performance when some movement was involved, for example in a police shooting simulator (Saus et al., 2006) or with a navigation simulator (Saus et al., 2012). This is encouraging for future studies aimed to reproduce ecologically valid situations with HRV.

### Respiration

Controlling for respiration is a long debate within HRV research. The proposed reason for controlling respiration is that HRV could be affected in certain circumstances by respiratory depth, the amount of air taken into the lungs (Hirsch and Bishop, 1981), and respiratory frequency, the amount of breaths per minute (Brown et al., 1993; Houtveen et al., 2002). It could also be affected by the central respiratory drive, estimated through partial pressure of CO_2_ (Houtveen et al., 2002). Hence, in order to accurately assess vagal function, it has been proposed to “correct” HRV for respiration (Grossman, 1992). Thus it has been proposed that these respiratory factors require experimental controls either online during the experiment or offline after the experiment with *post hoc* statistical analyses. However, the routine control of respiration is problematic for several reasons that we and others have described in detail (Denver et al., 2007; Larsen et al., 2010; Thayer et al., 2011; Lewis et al., 2012). Briefly, researchers (Larsen et al., 2010; Thayer et al., 2011; Dick et al., 2014) suggest a common basis for HRV and respiration, with a bi-directional communication between the respiratory and cardiovascular systems (Thayer et al., 2012). Therefore this would deem a routine correction of HRV for respiration problematic. However, we will detail some of the issues associated with respiration and HRV below.

Regarding respiratory depth, respiratory sinus arrhythmia [which reflects HF when the breathing frequency is comprised between 9 and 24 cycles per minute (Malik, 1996)] shows greater amplitude during higher tidal volumes and lower respiratory frequencies (Hirsch and Bishop, 1981; Shaffer et al., 2014). Respiratory depth is linked to tidal volume and controlling for tidal volume can be done with pneumotachography (Quintana and Heathers, 2014). This process allows for tidal volume to be measured, however, this process requires a face mask which may not be practical to use and might create interferences in experimental psychophysiological research. A *post hoc* approach could be to use a dedicated algorithm to control for tidal volume (e.g., Schulz et al., 2009). However, the effect of respiratory depth or tidal volume on HRV has been shown to account for less than 5% of the variance in the several measures of HRV but more than 10% of the variance when using the peak-to-trough method (Lewis et al., 2012).

Regarding respiratory frequency, one component that may be heavily influenced by it is vagal tone because HF is deemed to reflect vagal tone only when breathing frequency is higher than nine cycles per minute (Malik, 1996; Berntson et al., 1997). More specifically, HF corresponds to vagal tone when between 0.15 and 0.40 Hz, which means between 9 and 24 cycles per minute regarding respiratory rate. Therefore, any respiratory rate below or above this interval HF may not accurately depict vagal tone anymore. In comparison to HF, RMSSD has been shown to be less affected by respiratory rate (Hill and Siebenbrock, 2009). Thus, we still need to have knowledge of the respiratory rate in order to determine whether the changes we observe in HRV values are primarily due to changes in respiratory frequency (Kuehl et al., 2015).

It is possible to control online and offline for respiratory rate. Doing this online would require using a strain gage during the experiment (Quintana and Heathers, 2014). When this is not possible, and considering the ambiguity of some strain gage estimates (Thayer et al., 2002), there is still an option to control this offline with a *post hoc* estimation. An estimate of respiratory rate can for example be derived from the central frequency of the HF component detected in an autoregressive analysis of HR. The central frequency of HF-HRV is highly correlated with strain gage measures of respiration (Thayer et al., 2002). However, the limitation of this method is that there should be an observable HF component. If there is no observable peak, it is questionable whether there is really any true HF power, or if it is just noise. This is one reason why the autoregressive analysis of HR is preferred to Fast Fourier Transform in this case (Thayer et al., 2002). Another estimation method, originally developed by Moody et al. (1985), is available with Kubios to estimate from ECG data the respiratory frequency from changes in R-wave amplitude, which is called the ECG derived respiration (Tarvainen et al., 2014). As a conclusion regarding the measure of respiratory rate, a strain gage would provide more accurate results given it would account for any non-cyclical respiration patterns (e.g., sudden sighs, coughs). However, if no strain gage is available, the offline methods based on the calculation of the HF peak obtained with autoregressive analysis or on the ECG derived respiration could be used.

Another approach would be to force participants to breathe at a specific rate during the experiment (Grossman and Taylor, 2007). A compromise could be to control for respiration rate during the experiment through measuring a participant’s natural breathing rate, and then using the derived frequency for respiratory pacing (Elstad, 2012). While these approaches could still work for baseline measurement, this procedure may influence HRV during emotional or cognitive task. If the participant has to consciously follow the pacing cue, in addition to paying attention to the experimental task, this might then influence task output (Quintana and Heathers, 2014). However, the effect of paced breathing, even at the pace of spontaneous breathing, is problematic as it has been shown to either increase, decrease, or not change estimates of HF HRV in a manner that is not predictable (Larsen et al., 2010); while in some cases paced breathing provides similar results to spontaneous breathing (e.g., Bertsch et al., 2012).

A totally different approach is to let participants breathe spontaneously because forcing participants to breathe at a specific pace would suppress an important influence on HRV (Denver et al., 2007). As reviewed by Thayer et al. (2011), there is an aggregate of evidence from behavior genetics, neuroimaging, cardiorespiratory coupling, and psychophysiological studies suggesting that the removal of variance associated with respiration from HRV would remove variance associated with the common neural origin of HRV and respiration.

Whereas there is continued controversy regarding respiratory control, several things are clear: (1) different indices of HRV are differentially affected by breathing with the peak-to-trough method being most affected (e.g., Penttila et al., 2001; Hill et al., 2009; Lewis et al., 2012); (2) when analyzed appropriately, even measures derived from the peak-to-trough method can be reliable indicators of HRV without additional respiratory control (Lewis et al., 2012); (3) it has been repeatedly shown, that the effects of respiration on parasympathetic indices of HRV when recorded under resting state conditions are minimal at best – and resting state HRV is recorded best under conditions of spontaneous breathing (e.g., Larsen et al., 2010; Bertsch et al., 2012). Controlling for respiration when examining HRV indices will remove variability associated with neural control over the heartbeat, and therefore some of the variance that the researcher is actually interested in would be removed (see Larsen et al., 2010 for a thorough review); (4) it is useful to have some indication of respiration to aid the interpretation of HRV and to ascertain that participants were breathing “normally.”

In sum, based on the most recent evidence on this topic (Thayer et al., 2011), we recommend researchers do not engage in routine correction of HRV for respiration in case of spontaneous breathing. However, we still recommend monitoring respiration, in order to foster the understanding of the neurobiological mechanisms and contextual factors responsible for the complex interactions between the respiratory and cardiovascular system. Additionally, researchers should check whether respiratory frequency remains between 9 and 24 cycles per minute (corresponding to the HF band, 0.15–0.40 Hz). If conclusions have to be made regarding vagal tone it is important to have no differences in respiratory frequency between experimental tasks or between case and control groups.

## HRV Data Analysis

In this section we will provide recommendations regarding HRV software, artifact correction, normality of HRV data, HRV frequency-domain analysis, and which HRV variables to analyze.

### HRV Software

The analysis of HRV data has been made very accessible through a free popular software, Kubios (Tarvainen et al., 2014), which is currently the most used by researchers. New softwares like gHRV (Rodriguez-Linares et al., 2014), a package for the R statistical environment, and ARTiiFACT (Kaufmann et al., 2011) are in development and offer other analysis tools such as different analysis options, visualize and export the HRV data, as well as the possibility to edit the source code for gHRV. Having access to the original source code is of value when comparing it to proprietary software, as the source code can be used to interpret what is being measured.

### Artifact Correction

Any HRV data sets requires a signal pre-processing before proceeding to the analysis, with the objective to identify the fiducial points (typically the R peak) from a normal ECG QRS complex. Hence, all abnormal beats not generated by sinus node depolarisations should be eliminated from the record. HRV data from ambulatory recordings generally contain more artifacts that can be either of physiological or technical origins. Technical artifacts may result from poorly attached electrodes or to excessive motion from the individual. Physiological artifacts may include ectopic beats, atrial fibrillations sighs and coughs.

When recording the IBI this only allows you the possibility to carry out an automatic artifact correction, given the fiducial points of an ECG are not recorded. For example Kubios will allow you to automatically filter your data, the purpose of this is to detect RR intervals that differ “abnormally” from the normal mean RR interval which may represent an artifact (Tarvainen and Niskanen, 2012). The different threshold levels for artifact correction in Kubios are the following: very low = 0.45 s, low = 0.35 s, medium = 0.25 s, strong = 0.15 s, very strong = 0.05 s. This procedure is very commonly used and considered sufficient in most cases for example when data was recorded in good conditions and little movement or electrode/belt movements. However, we would strongly advise to record ECG signal because in doing this the researcher is able to edit the data and modify the artifact correction manually afterward by performing a visual inspection of the ECG signal. We therefore highly recommend not to rely only on an automatic artifact correction like the one offered by Kubios, because artifacts detected by the automatic procedure of Kubios may correspond to real heartbeats, as displayed in the Supplementary Materials (see Data Sheet 3), and recommend instead to visually inspecting the ECG signal. The consequence of deleting a real heart beat that is assumed to be an artifact may have critical consequences. These consequences include a substantial influence on the HRV values and losing precious information regarding the variability of the heart rate signal. In the Supplementary Material example we see that using the automatic correction option of Kubios (very low filter) could lead to an 11% error rate in the evaluation of vagal tone. This is eloquently explained by Berntson and Stowell (1998) who stated that only one heartbeat makes a difference in the analysis. Additional software can assist you in the detection of the artifacts seen on ECG signals, like ArtIifact (Kaufmann et al., 2011). Finally, we can refer the reader to a useful guide on how to prepare the HRV recording prior to the analysis (Shaffer and Combatalade, 2013). As a last remark, in case the experiment involved different conditions it would be advantageous that the person handling the HRV data, particularly when performing the artifact analysis, is unaware of the experimental conditions. This reduces experimenter bias and reduces the possibility of investigating for particular or suggestive results.

### Normality of HRV Parameters

In many studies we observe a non-normal distribution of HRV parameters. In this case it is necessary to proceed to data transformation prior to their analysis, a common procedure is to log transform the data to adjust for the unequal variance and many studies report for example the natural logarithm of RMSSD (Stanley et al., 2013b) or the natural logarithm of the power values in ms^2^ (e.g., Prinsloo et al., 2011).

### Frequency Analysis of HRV Parameters

Regarding the frequency domain the researcher is faced with variables presented with different units. In line with the Task Force (Malik, 1996), we would always recommend to present the absolute power and the normalized units even though there can be some problems using normalized units (Heathers, 2014). These normalized units represent the relative value of each power component in proportion to the total power minus the VLF component.

Another choice faced by the researcher in frequency analysis is to decide between two main frequency analysis methods: whether to use Fast Fourier Transform or autoregressive modeling. Both analyses techniques usually correlate highly (between *r* = 0.86 and *r* = 0.91) in the HF band (Hayano et al., 1991). Fast Fourier Transform has been one of the most utilized techniques so far but AR is gaining interest. In regards to the visual display of data, the AR demonstrated better resolution of sharp peaks than FFT, and makes a smoother, more interpretable curve (Burr and Cowan, 1992; Cowan et al., 1992). Moreover, several authors observed that FFT overestimated the HF component, compared with autoregressive analysis (Badilini et al., 1988; Fagard et al., 1998; Pichon et al., 2006). Therefore we would recommend focusing on autoregressive analysis for HF band calculation. The model-order chosen to perform AR should always be indicated (Malik, 1996) and be no shorter than 16 for short-term recordings (Boardman et al., 2002).

### HRV Variables to Analyze

If researchers choose to conduct a psychophysiological research project based on one of the five theories we presented in section “HRV in Psychophysiological Research: A Focus on Vagal Tone,” they will be interested in assessing vagal tone. If the researcher is aiming to identify vagal tone, our recommendation is to analyze one of the main variables reflecting it (i.e., either RMSSD, peak-valley, or HF). Moreover, to avoid bias within the research, we would recommend in addition to the main analysis performed with one variable reflecting vagal tone, to performing as well the same analyses with the other variables depicting vagal tone, to check whether results echo the findings across variables supposed to reflect vagal tone.

### HRV Data Reporting

Recent guidelines regarding the reporting of HRV studies were introduced by Quintana et al. (2016) and insist on the need to consistently report key experimental elements in order to advance of the field. The guidelines include four main elements: participant selection, IBI collection, IBI analysis and cleaning, and HRV calculation. They came up with an easy-to-follow 13-items checklist that will prove to be very useful for psychophysiological researchers, even though the guidelines focused on psychiatry. Given the extensive description of those guidelines concerning the reporting of HRV studies, we won’t elaborate in details here on this topic, and instead refer the reader to this very informative paper. However, we would just like to stress a crucial point regarding the specific reporting of HRV data in scientific papers: currently, readers may find themselves frustrated when reading the result sections of HRV psychophysiological experiments because often the variables used to display vagal tone were not the same. This makes it extremely hard to compare results across studies as well as complicating the integration of findings into a comprehensive review or a meta-analysis, which subsequently hinders the development of the field. We understand that scientific journals have space restrictions and that every single HRV variable can’t be displayed in every table. However, as more and more journals allow now attaching files as supplementary online material, whenever possible we recommend researchers to update their HRV raw data files as well as the statistical analyses realized with the other HRV variables, ensuring previously that ethics committee and participants explicitly agreed to public data sharing, even in an anonymous form. In addition, if novel HRV methods are used, researchers should follow recent recommendations and always present the new HRV measures together to more traditional measures of HRV (Sassi et al., 2015). This will allow for many researchers to contribute to the development of HRV metrics and guidelines and subsequently develop the field.

## Conclusion

The aim of this paper was to provide the field of psychophysiology with practical recommendations concerning research conducted with HRV, specifically highlighting its ability to index cardiac vagal tone, which is relevant for many psychophysiological phenomena, such as self-regulation mechanisms linked to cognitive, affective, social, and health (Porges, 2007; Thayer et al., 2009; McCraty and Shaffer, 2015). These recommendations aimed to cover experiment planning, measurement standards, recommendations regarding data analysis and data reporting. Our endeavor here was not to strive to establish standards surrounding HRV, but rather to offer the reader with a comprehensive overview of the different issues to consider. Again, we could not be exhaustive and address in-depth all issues we talked about in this paper, but we always refer the reader to useful contributions that will help them make educated choices. This can be considered as a timely contribution as this paper falls two decades after the Task Force on HRV (Malik, 1996) and one decade after a major milestone driving HRV research psychophysiology, the special issue on cardiac vagal tone edited by Chambers and Allen (2007), and completes recent overview works on HRV research (Quintana and Heathers, 2014; Shaffer et al., 2014; Sassi et al., 2015; Quintana et al., 2016). A summary of the recommendations is presented in **Table 2**. Again these recommendations should not be considered as imperative guidelines to follow, given of the breadth of potential research questions to be addressed. Instead they are rather thought to accompany the researcher in psychophysiology and to ease the use of HRV to investigate phenomena related to self-regulation at the cognitive, emotional, social and health level. Our recommendations are also aimed to guide researchers through complicated interpretation that follows HRV data collection, which is often clouded by methodological concerns. As a result we endeavored to cover the relevant areas concerning HRV research within psychophysiology, with a focus on vagal tone given its theoretical relevance, contributing to make HRV one of the pillars of psychophysiological research in the 21st century.

**Table 2:** Summary of recommendations for heart rate variability assessment – with a focus on cardiac vagal tone – for psychophysiological research – Experiment planning, data analysis, and data reporting.

	Parameter	Recommendations
Experiment planning	HRV variables to assess	A focus on vagal tone is recommended, vagal tone being measured through: RMSSD and pNN50 in the time-domain, and HF in the frequency-domain; additional parameters potentially of interest according to research question
	Within-subject design vs. between subject design	Within-subject design
	Sample size	Rule-of-thumb of 233, 61, and 21 participants to detect, respectively, small, medium, and large effect sizes, always accompanied by a power analysis using for example G^∗^power 3
	Experiment structure	Three Rs: resting, reactivity, recovery
	Variables to control – stable and transient	See Supplementary Materials (Data Sheet 1)
	HRV as dependent or independent variable	Depends on research question
Measurement standards	Baseline recording	Sitting, knees with a 90° angle, hands on thighs, eyes closed
	Measurement in ambulatory settings	Possible but need to control for respiration and physical activity
	Movement	For clear interpretation of psychophysiological phenomena, and specifically vagal tone: no movement
	HRV recording device and signal	Device using electrodes and allowing to record ECG signal, only IBI is not precise enough for artifact correction
	ECG sampling	Minimum 125 Hz, 500 Hz being seen as a conservative guideline
	Duration of recording	Five minutes when possible to enable comparison between clinical studies, otherwise shorter recordings can be envisaged depending on research question. At least 1 min for when vagal tone is targeted with frequency analysis, ultra-short analysis for vagal tone possible with time-domain analysis (shorter possible according to research question), 24 h for long-term recordings; don’t compare recordings of different lengths.
	Respiration	Do not systematically control for it (may bias the interpretation) – but assess it
Data analysis	Software	Any software allowing editing of the ECG signal (for example Kubios)
	Artifact correction	Based on ECG signal, manual or assisted by specific software
	Non-normally distributed HRV parameters	Data transformation with natural logarithm
	Frequency-domain variables: absolute power, %, normalized units	Always report at least absolute power
	Frequency-domain analysis method	Autoregressive modeling (AR) should be preferred to Fast Fourier Transform (FFT)
	HRV variables to analyze	If research question is based on vagal tone: perform the analyses with one main variable indexing vagal tone (e.g., RMSSD, peak-valley, and HF); perform same analyses with the other variables depicting vagal tone to check whether results are consistent
Data reporting	HRV variables to report	In the paper present one main variable illustrating vagal tone for comprehension purposes (e.g., RMSSD, peak-valley, HF AR, and HF FFT); then submit as Supplementary Material all raw data as well as the analysis ran with the other HRV parameters to contribute to the development of HRV metrics and guidelines as well as the analyses ran with the other HRV parameters

## Author Contributions

SL prepared the first draft, EM and JT provided insightful comments that critically improved the manuscript quality.

## Conflict of Interest Statement

The authors declare that the research was conducted in the absence of any commercial or financial relationships that could be construed as a potential conflict of interest.

## References

[B1] Alvares G. A. Quintana D. S. Hickie I. B. Guastella A. J. (2016). Autonomic nervous system dysfunction in psychiatric disorders and the impact of psychotropic medications: a systematic review and meta-analysis. *J. Psychiatry Neurosci.* 41 89–104. 10.1503/jpn.140217 26447819 PMC4764485

[B2] Badilini F. Maison-Blanche P. Coumel P. (1988). Heart rate variability in passive tilt test: comparative evaluation of autoregressive and FFT spectral analyses. *Pacing Clin. Electrophysiol.* 21 1122–1132. 10.1111/j.1540-8159.1998.tb00159.x 9604245

[B3] Berna G. Ott L. Nandrino J. L. (2014). Effects of emotion regulation difficulties on the tonic and phasic cardiac autonomic response. *PLoS ONE* 9:e102971 10.1371/journal.pone.0102971 PMC4108383 25054913

[B4] Berntson G. G. Bigger J. T. Eckberg D. L. Grossman P. Kaufmann P. G. Malik M. (1997). Heart rate variability: origins, methods, and interpretive caveats. *Psychophysiology* 34 623–648. 9401419 10.1111/j.1469-8986.1997.tb02140.x

[B5] Berntson G. G. Quigley K. S. Lozano D. (2007). “Cardiovascular psychophysiology,” in *Handbook of Psychophysiology*, eds Cacioppo J. T. Tassinary L. G. Berntson G. G. (New York, NY: Cambridge University Press).

[B6] Berntson G. G. Stowell J. R. (1998). ECG artifacts and heart period variability: dont miss a beat! *Psychophysiology* 35 127–132. 10.1111/1469-8986.3510127 9499713

[B7] Bertsch K. Hagemann D. Naumann E. Schachinger H. Schulz A. (2012). Stability of heart rate variability indices reflecting parasympathetic activity. *Psychophysiology* 49 672–682. 10.1111/j.1469-8986.2011.01341.x 22335779

[B8] Billman G. E. (2013). The LF/HF ratio does not accurately measure cardiac sympatho-vagal balance. *Front. Physiol.* 4:26 10.3389/fphys.2013.00026 PMC3576706 23431279

[B9] Billman G. E. Huikuri H. V. Sacha J. Trimmel K. (2015). An introduction to heart rate variability: methodological considerations and clinical applications. *Front. Physiol.* 6:55 10.3389/fphys.2015.00055 PMC4340167 25762937

[B10] Boardman A. Schlindwein F. S. Rocha A. P. Leite A. (2002). A study on the optimum order of autoregressive models for heart rate variability. *Physiol. Meas.* 23 325–336. 12051304 10.1088/0967-3334/23/2/308

[B11] Bravi A. Longtin A. Seely A. J. (2011). Review and classification of variability analysis techniques with clinical applications. *Biomed. Eng. Online* 10:90 10.1186/1475-925x-10-90 PMC3224455 21985357

[B12] Brodal P. (2010). *The Central Nervous System – Structure and Function.* New York, NY: Oxford University Press.

[B13] Brown T. E. Beightol L. A. Koh J. Eckberg D. L. (1993). Important influence of respiration on human R-R interval power spectra is largely ignored. *J. Appl. Physiol.* (1985) 75 2310–2317. 8307890 10.1152/jappl.1993.75.5.2310

[B14] Buchheit M. Simon C. Charloux A. Doutreleau S. Piquard F. Brandenberger G. (2005). Heart rate variability and intensity of habitual physical activity in middle-aged persons. *Med. Sci. Sports Exerc.* 37 1530–1534. 10.1249/01.mss.0000177556.05081.77 16177605

[B15] Burr R. L. Cowan M. J. (1992). Autoregressive spectral models of heart rate variability. Practical issues. *J. Electrocardiol.* 25(Suppl.), 224–233. 1297702 10.1016/0022-0736(92)90108-c

[B16] Cervantes Blásquez J. C. Rodas Font G. Capdevila Ortís L. (2009). Heart-rate variability and precompetitive anxiety in swimmers. *Psicothema* 21 531–536. 19861094

[B17] Chambers A. S. Allen J. J. B. (2007). Cardiac vagal control, emotion, psychopathology, and health. *Biol. Psychol.* 74 113–115. 10.1016/j.biopsycho.2006.09.004 17055143

[B18] Champely S. (2016). *pwr: Basic Functions for Power Analysis.* Available at: http://CRAN.R-project.org/package=pwr

[B19] Chapleau M. W. Sabharwal R. (2011). Methods of assessing vagus nerve activity and reflexes. *Heart Fail. Rev.* 16 109–127. 10.1007/s10741-010-9174-6 20577901 PMC4322860

[B20] Cohen D. (1988). *Statistical Power Analysis for Behavioral Sciences.* Hillsdale, MI: Erlbaum.

[B21] Cohen H. Loewenthal U. Matar M. Kotler M. (2001). Association of autonomic dysfunction and clozapine. Heart rate variability and risk for sudden death in patients with schizophrenia on long-term psychotropic medication. *Br. J. Psychiatry* 179 167–171. 11483480 10.1192/bjp.179.2.167

[B22] Collins S. Karasek R. (2010). Reduced vagal cardiac control variance in exhausted and high strain job subjects. *Int. J. Occup. Med. Environ. Health* 23 267–278. 10.2478/v10001-010-0023-6 20934956

[B23] Cowan M. J. Burr R. L. Narayanan S. B. Buzaitis A. Strasser M. Busch S. (1992). Comparison of autoregression and fast Fourier transform techniques for power spectral analysis of heart period variability of persons with sudden cardiac arrest before and after therapy to increase heart period variability. *J. Electrocardiol.* 25(Suppl.), 234–239. 1297703 10.1016/0022-0736(92)90109-d

[B24] Denver J. W. Reed S. F. Porges S. W. (2007). Methodological issues in the quantification of respiratory sinus arrhythmia. *Biol. Psychol.* 74 286–294. 10.1016/j.biopsycho.2005.09.005 17067734 PMC1828207

[B25] Dick T. E. Hsieh Y. H. Dhingra R. R. Baekey D. M. Galan R. F. Wehrwein E. (2014). Cardiorespiratory coupling: common rhythms in cardiac, sympathetic, and respiratory activities. *Prog. Brain Res.* 209 191–205. 10.1016/B978-0-444-63274-6.00010-2 24746049 PMC4052709

[B26] Duschek S. Muckenthaler M. Werner N. Reyes del Paso G. A. (2009). Relationships between features of autonomic cardiovascular control and cognitive performance. *Biol. Psychol.* 81 110–117. 10.1016/j.biopsycho.2009.03.003 19428975

[B27] Eckberg D. L. (1983). Human sinus arrhythmia as an index of vagal cardiac outflow. *J. Appl. Physiol. Respir. Environ. Exerc. Physiol.* 54 961–966. 6853303 10.1152/jappl.1983.54.4.961

[B28] Eckberg D. L. (1997). Sympathovagal balance: a critical appraisal. *Circulation* 96 3224–3232. 9386196 10.1161/01.cir.96.9.3224

[B29] Eckberg D. L. Eckberg M. J. (1982). Human sinus node responses to repetitive, ramped carotid baroreceptor stimuli. *Am. J. Physiol.* 242 H638–H644. 7065276 10.1152/ajpheart.1982.242.4.H638

[B30] Egizio V. B. Jennings J. R. Christie I. C. Sheu L. K. Matthews K. A. Gianaros P. J. (2008). Cardiac vagal activity during psychological stress varies with social functioning in older women. *Psychophysiology* 45 1046–1054. 10.1111/j.1469-8986.2008.00698.x 18823424 PMC2866176

[B31] Ellis R. J. Zhu B. Koenig J. Thayer J. F. Wang Y. (2015). A careful look at ECG sampling frequency and R-peak interpolation on short-term measures of heart rate variability. *Physiol. Meas.* 36 1827–1852. 10.1088/0967-3334/36/9/1827 26234196

[B32] Elstad M. (2012). Respiratory variations in pulmonary and systemic blood flow in healthy humans. *Acta Physiol.* 205 341–348. 10.1111/j.1748-1716.2012.02419.x 22289157

[B33] Esco M. R. Flatt A. A. (2014). Ultra-short-term heart rate variability indexes at rest and post-exercise in athletes: evaluating the agreement with accepted recommendations. *J. Sports Sci. Med.* 13 535–541. 25177179 PMC4126289

[B34] Fagard R. H. Pardaens K. Staessen J. A. Thijs L. (1998). Power spectral analysis of heart rate variability by autoregressive modelling and fast Fourier transform: a comparative study. *Acta Cardiol.* 53 211–218. 9842406

[B35] Faul F. Erdfelder E. Buchner A. Lang A. G. (2009). Statistical power analyses using G^∗^Power 3.1: tests for correlation and regression analyses. *Behav. Res. Methods* 41 1149–1160. 10.3758/BRM.41.4.1149 19897823

[B36] Faul F. Erdfelder E. Lang A. G. Buchner A. (2007). G^∗^Power 3: a flexible statistical power analysis program for the social, behavioral, and biomedical sciences. *Behav. Res. Methods* 39 175–191. 17695343 10.3758/bf03193146

[B37] Fenton-O’Creevy M. Lins J. (2012). Emotion regulation and trader expertise: heart rate variability on the trading floor. *J. Neurosci. Psychol. Econ.* 5 227–237. 10.1037/a0030364

[B38] Flatt A. A. Esco M. R. (2013). Validity of the ithlete^TM^ smart phone application for determining ultra-short-term heart rate variability. *J. Hum. Kinet.* 39 85–92. 10.2478/hukin-2013-0071 24511344 PMC3916914

[B39] Ghuman N. Campbell P. White W. B. (2009). Role of ambulatory and home blood pressure recording in clinical practice. *Curr. Cardiol. Rep.* 11 414–421. 19863865 10.1007/s11886-009-0060-6 PMC2896788

[B40] Gil E. Orini M. Bailon R. Vergara J. M. Mainardi L. Laguna P. (2010). Photoplethysmography pulse rate variability as a surrogate measurement of heart rate variability during non-stationary conditions. *Physiol. Meas.* 31 1271–1290. 10.1088/0967-3334/31/9/015 20702919

[B41] Grossman P. (1992). Respiratory and cardiac rhythms as windows to central and autonomic biobehavioral regulation: selection of window frames, keeping the panes clean and viewing the neural topography. *Biol. Psychol.* 34 131–161. 1467391 10.1016/0301-0511(92)90013-k

[B42] Grossman P. Taylor E. W. (2007). Toward understanding respiratory sinus arrhythmia: relations to cardiac vagal tone, evolution and biobehavioral functions. *Biol. Psychol.* 74 263–285. 10.1016/j.biopsycho.2005.11.014 17081672

[B43] Grossman P. van Beek J. Wientjes C. (1990). A comparison of three quantification methods for estimation of respiratory sinus arrhythmia. *Psychophysiology* 27 702–714. 2100356 10.1111/j.1469-8986.1990.tb03198.x

[B44] Grossman P. Wilhelm F. H. Spoerle M. (2004). Respiratory sinus arrhythmia, cardiac vagal control, and daily activity. *Am. J. Physiol. Heart Circ. Physiol.* 287 H728–H734. 10.1152/ajpheart.00825.2003 14751862

[B45] Hansen A. L. Johnsen B. H. Thayer J. F. (2003). Vagal influence on working memory and attention. *Int. J. Psychophysiol.* 48 263–274. 10.1016/S0167-8760(03)00073-4 12798986

[B46] Hayano J. Sakakibara Y. Yamada A. Yamada M. Mukai S. Fujinami T. (1991). Accuracy of assessment of cardiac vagal tone by heart rate variability in normal subjects. *Am. J. Cardiol.* 67 199–204. 1987723 10.1016/0002-9149(91)90445-q

[B47] Hayano J. Yamada M. Sakakibara Y. Fujinami T. Yokoyama K. Watanabe Y. (1990). Short- and long-term effects of cigarette smoking on heart rate variability. *Am. J. Cardiol.* 65 84–88. 2294686 10.1016/0002-9149(90)90030-5

[B48] Heathers J. A. (2014). Everything Hertz: methodological issues in short-term frequency-domain HRV. *Front. Physiol.* 5:177 10.3389/fphys.2014.00177 PMC4019878 24847279

[B49] Hill L. K. Siebenbrock A. (2009). Are all measures created equal? Heart rate variability and respiration. *Biomed. Sci. Instrum.* 45 71–76. 19369742

[B50] Hill L. K. Siebenbrock A. Sollers J. J. Thayer J. F. (2009). Are all measures created equal? Heart rate variability and respiration – biomed 2009. *Biomed. Sci. Instrum.* 45 71–76. 19369742

[B51] Hirsch J. A. Bishop B. (1981). Respiratory sinus arrhythmia in humans: how breathing pattern modulates heart rate. *Am. J. Physiol.* 241 H620–H629. 7315987 10.1152/ajpheart.1981.241.4.H620

[B52] Houtveen J. H. Rietveld S. de Geus E. J. (2002). Contribution of tonic vagal modulation of heart rate, central respiratory drive, respiratory depth, and respiratory frequency to respiratory sinus arrhythmia during mental stress and physical exercise. *Psychophysiology* 39 427–436. 12212635 10.1017/S0048577202394022

[B53] Inoue N. Kuroda K. Sugimoto A. Kakuda T. Fushiki T. (2003). Autonomic nervous responses according to preference for the odor of jasmine tea. *Biosci. Biotechnol. Biochem.* 67 1206–1214. 10.1271/bbb.67.1206 12843644

[B54] Jarczok M. N. Li J. Mauss D. Fischer J. E. Thayer J. F. (2012). Heart rate variability is associated with glycemic status after controlling for components of the metabolic syndrome. *Int. J. Cardiol.* 167 855–861. 10.1016/j.ijcard.2012.02.002 22386703

[B55] Jennings J. R. Allen B. Gianaros P. J. Thayer J. F. Manuck S. B. (2015). Focusing neurovisceral integration: cognition, heart rate variability, and cerebral blood flow. *Psychophysiology* 52 214–224. 10.1111/psyp.12319 25160649 PMC4387874

[B56] Jennings J. R. Kamarck T. Stewart C. Eddy M. Johnson P. (1992). Alternate cardiovascular baseline assessment techniques: vanilla or resting baseline. *Psychophysiology* 29 742–750. 1461961 10.1111/j.1469-8986.1992.tb02052.x

[B57] Johnsen B. H. Thayer J. F. Laberg J. C. Wormnes B. Raadal M. Skaret E. (2003). Attentional and physiological characteristics of patients with dental anxiety. *J. Anxiety Disord.* 17 75–87. 12464290 10.1016/s0887-6185(02)00178-0

[B58] Kaufmann T. Sütterlin S. Schulz S. M. Vögele C. (2011). ARTiiFACT: a tool for heart rate artifact processing and heart rate variability analysis. *Behav. Res. Methods* 43 1161–1170. 10.3758/s13428-011-0107-7 21573720

[B59] Kemp A. H. Quintana D. S. Gray M. A. Felmingham K. L. Brown K. Gatt J. M. (2010). Impact of depression and antidepressant treatment on heart rate variability: a review and meta-analysis. *Biol. Psychiatry* 67 1067–1074. 10.1016/j.biopsych.2009.12.012 20138254

[B60] Kirschbaum C. Kudielka B. M. Gaab J. Schommer N. C. Hellhammer D. H. (1999). Impact of gender, menstrual cycle phase, and oral contraceptives on the activity of the hypothalamus-pituitary-adrenal axis. *Psychosom. Med.* 61 154–162. 10204967 10.1097/00006842-199903000-00006

[B61] Kleiger R. E. Stein D. S. Bigger M. D. (2005). Heart rate variability: measurement and clinical utility. *Ann. Noninvasive Electrocardiol.* 10 88–101. 10.1111/j.1542-474X.2005.10101.x 15649244 PMC6932537

[B62] Kligfield P. Gettes L. S. Bailey J. J. Childers R. Deal B. J. Hancock E. (2007). Recommendations for the standardization and interpretation of the electrocardiogram: part I: the electrocardiogram and its technology a scientific statement from the American Heart Association Electrocardiography and Arrhythmias Committee, Council on Clinical Cardiology; the American College of Cardiology Foundation; and the Heart Rhythm Society endorsed by the International Society for Computerized Electrocardiology. *J. Am. Coll. Cardiol.* 49 1109–1127. 10.1016/j.jacc.2007.01.024 17349896

[B63] Kuehl L. K. Deuter C. E. Richter S. Schulz A. Ruddel H. Schachinger H. (2015). Two separable mechanisms are responsible for mental stress effects on high frequency heart rate variability: an intra-individual approach in a healthy and a diabetic sample. *Int. J. Psychophysiol.* 95 299–303. 10.1016/j.ijpsycho.2014.12.003 25500224

[B64] Laborde S. Brüll A. Weber J. Anders L. S. (2011). Trait emotional intelligence in sports: a protective role against stress through heart rate variability? *Pers. Individ. Dif.* 51 23–27. 10.1016/j.paid.2011.03.003

[B65] Laborde S. Furley P. Schempp C. (2015a). The relationship between working memory, reinvestment, and heart rate variability. *Physiol. Behav.* 139 430–436. 10.1016/j.physbeh.2014.11.036 25449388

[B66] Laborde S. Lautenbach F. Allen M. S. (2015b). The contribution of coping-related variables and heart rate variability to visual search performance under pressure. *Physiol. Behav.* 139 532–540. 10.1016/j.physbeh.2014.12.003 25481358

[B67] Laborde S. Raab M. (2013). The tale of hearts and reason: the influence of mood on decision making. *J. Sport Exerc. Psychol.* 35 339–357. 23966445 10.1123/jsep.35.4.339

[B68] Laborde S. Raab M. Kinrade N. P. (2014). Is the ability to keep your mind sharp under pressure reflected in your heart? Evidence for the neurophysiological bases of decision reinvestment. *Biol. Psychol.* 100 34–42. 10.1016/j.biopsycho.2014.05.003 24859424

[B69] Larsen P. D. Tzeng Y. C. Sin P. Y. Galletly D. C. (2010). Respiratory sinus arrhythmia in conscious humans during spontaneous respiration. *Respir. Physiol. Neurobiol.* 174 111–118. 10.1016/j.resp.2010.04.021 20420940

[B70] Lehrer P. M. (2013). How does heart rate variability biofeedback work? resonance, the baroreflex, and other mechanisms. *Biofeedback* 41 26–31. 10.5298/1081-5937-41.1.02

[B71] Lewis G. F. Furman S. A. McCool M. F. Porges S. W. (2012). Statistical strategies to quantify respiratory sinus arrhythmia: Are commonly used metrics equivalent? *Biol. Psychol.* 89 349–364. 10.1016/j.biopsycho.2011.11.009 22138367 PMC3269511

[B72] Lewis M. J. Kingsley M. Short A. L. Simpson K. (2007). Rate of reduction of heart rate variability during exercise as an index of physical work capacity. *Scand. J. Med. Sci. Sports* 17 696–702. 10.1111/j.1600-0838.2006.00616.x 17346290

[B73] Lu C. L. Zou X. Orr W. C. Chen J. D. (1999). Postprandial changes of sympathovagal balance measured by heart rate variability. *Dig. Dis. Sci.* 44 857–861. 10219849 10.1023/a:1026698800742

[B74] Malik M. (1996). Heart rate variability. Standards of measurement, physiological interpretation, and clinical use. Task Force of the European Society of Cardiology and the North American Society of Pacing and Electrophysiology. *Eur. Heart J.* 17 354–381. 8737210

[B75] Marcovitch S. Leigh J. Calkins S. D. Leerks E. M. O’Brien M. Blankson A. N. (2010). Moderate vagal withdrawal in 3.5-year-old children is associated with optimal performance on executive function tasks. *Dev. Psychobiol.* 52 603–608. 10.1002/dev.20462 20806334 PMC3004152

[B76] Martin C. K. Heilbronn L. K. de Jonge L. DeLany J. P. Volaufova J. Anton S. (2007). Effect of calorie restriction on resting metabolic rate and spontaneous physical activity. *Obesity (Silver Spring)* 15 2964–2973. 10.1038/oby.2007.354 18198305

[B77] Massin M. M. Maeyns K. Withofs N. Ravet F. Gerard P. (2000). Circadian rhythm of heart rate and heart rate variability. *Arch. Dis. Child.* 83 179–182. 10906034 10.1136/adc.83.2.179 PMC1718415

[B78] Mateo M. Blasco-Lafarga C. Martínez-Navarro I. Guzmán J. F. Zabala M. (2012). Heart rate variability and pre-competitive anxiety in BMX discipline. *Eur. J. Appl. Physiol.* 112 113–123. 10.1007/s00421-011-1962-8 21503698

[B79] McCraty R. Childre D. (2010). Coherence: bridging personal, social, and global health. *Altern. Ther. Health Med.* 16 10–24. 20653292

[B80] McCraty R. Shaffer F. (2015). Heart rate variability: new perspectives on physiological mechanisms, assessment of self-regulatory capacity, and health risk. *Glob. Adv. Health Med.* 4 46–61. 10.7453/gahmj.2014.073 PMC4311559 25694852

[B81] Melillo P. Bracale M. Pecchia L. (2011). Nonlinear heart rate variability features for real-life stress detection. Case study: students under stress due to university examination. *Biomed. Eng. Online* 10:96 10.1186/1475-925X-10-96 PMC3305918 22059697

[B82] Messerotti Benvenuti S. Mennella R. Buodo G. Palomba D. (2015). Dysphoria is associated with reduced cardiac vagal withdrawal during the imagery of pleasant scripts: evidence for the positive attenuation hypothesis. *Biol. Psychol.* 106 28–38. 10.1016/j.biopsycho.2014.11.017 25643860

[B83] Miyake A. Friedman N. P. Emerson M. J. Witzki A. H. Howerter A. Wager T. D. (2000). The unity and diversity of executive functions and their contributions to complex “Frontal Lobe” tasks: a latent variable analysis. *Cognit. Psychol.* 41 49–100. 10.1006/cogp.1999.0734 10945922

[B84] Moody G. B. Mark R. G. Zoccola A. Mantero S. (1985). Derivation of respiratory signals from multi-lead ECGs. *Comput. Cardiol.* 12 113–116.

[B85] Munoz M. L. van Roon A. Riese H. Thio C. Oostenbroek E. Westrik I. (2015). Validity of (ultra-)short recordings for heart rate variability measurements. *PLoS ONE* 10:e0138921 10.1371/journal.pone.0138921 PMC4586373 26414314

[B86] Neijts M. Van Lien R. Kupper N. Boomsma D. Willemsen G. de Geus E. J. (2014). Heritability of cardiac vagal control in 24-h heart rate variability recordings: influence of ceiling effects at low heart rates. *Psychophysiology* 51 1023–1036. 10.1111/psyp.12246 24894483

[B87] Neumann S. A. Sollers J. J. Thayer J. F. Waldstein S. R. (2004). Alexithymia predicts attenuated autonomic reactivity, but prolonged recovery to anger recall in young women. *Int. J. Psychophysiol.* 53 183–195. 15246672 10.1016/j.ijpsycho.2004.03.008

[B88] Nisenbaum M. G. de Melo N. R. Giribela C. R. de Morais T. L. Guerra G. M. de Angelis K. (2014). Effects of a contraceptive containing drospirenone and ethinyl estradiol on blood pressure and autonomic tone: a prospective controlled clinical trial. *Eur. J. Obstet. Gynecol. Reprod. Biol.* 175 62–66. 10.1016/j.ejogrb.2014.01.006 24480113

[B89] Otzenberger H. Gronfier C. Simon C. Charloux A. Ehrhart J. Piquard F. (1998). Dynamic heart rate variability: a tool for exploring sympathovagal balance continuously during sleep in men. *Am. J. Physiol.* 275(3 Pt 2), H946–H950. 9724299 10.1152/ajpheart.1998.275.3.H946

[B90] Park G. Vasey M. W. Van Bavel J. J. Thayer J. F. (2014). When tonic cardiac vagal tone predicts changes in phasic vagal tone: the role of fear and perceptual load. *Psychophysiology* 51 419–426. 10.1111/psyp.12186 24571084

[B91] Penttila J. Helminen A. Jartti T. Kuusela T. Huikuri H. V. Tulppo M. (2001). Time domain, geometrical and frequency domain analysis of cardiac vagal outflow: effects of various respiratory patterns. *Clin. Physiol.* 21 365–376. 11380537 10.1046/j.1365-2281.2001.00337.x

[B92] Peschel S. K. Feeling N. R. Vogele C. Kaess M. Thayer J. F. Koenig J. (2016). A meta-analysis on resting state high-frequency heart rate variability in bulimia nervosa. *Eur. Eat. Disord. Rev.* 24 355–365. 10.1002/erv.2454 27241070

[B93] Pichon A. Roulaud M. Antoine-Jonville S. De Bisschop C. Denjean A. (2006). Spectral analysis of heart rate variability: Interchangeability between autoregressive analysis and fast Fourier transform. *J. Electrocardiol.* 39 31–37. 16387047 10.1016/j.jelectrocard.2005.08.001

[B94] Pickering T. G. White W. B. Giles T. D. Black H. R. Izzo J. L. Materson B. J. (2008). When and how to use self (home) and ambulatory blood pressure monitoring. *J. Am. Soc. Hypertens* 2 119–124. 10.1016/j.jash.2008.04.002 20409893

[B95] Piferi R. L. Kline K. A. Younger J. Lawler K. A. (2000). An alternative approach for achieving cardiovascular baseline: viewing an aquatic video. *Int. J. Psychophysiol.* 37 207–217. 10832007 10.1016/s0167-8760(00)00102-1

[B96] Piskorski J. Guzik P. (2005). Filtering Poincaré plots. *Comput. Methods Sci. Technol.* 11 39–48.

[B97] Porges S. W. (2007). The polyvagal perspective. *Biol. Psychol.* 74 116–143. 10.1016/j.biopsycho.2006.06.009 17049418 PMC1868418

[B98] Prinsloo G. E. Rauch H. G. Lambert M. I. Muench F. Noakes T. D. Derman W. E. (2011). The effect of short duration heart rate variability (HRV) biofeedback on cognitive performance during laboratory induced cognitive stress. *Appl. Cogn. Psychol.* 25 792–801. 10.1002/acp.1750

[B99] Quintana D. S. (2016). Statistical considerations for reporting and planning heart rate variability case-control studies. *Psychophysiology.* 10.1111/psyp.12798 [Epub ahead of print]. 27914167

[B100] Quintana D. S. Alvares G. A. Heathers J. A. (2016). Guidelines for reporting articles on psychiatry and heart rate variability (GRAPH): recommendations to advance research communication. *Transl. Psychiatry* 6:e803 10.1038/tp.2016.73 PMC5070064 27163204

[B101] Quintana D. S. Guastella A. J. McGregor I. S. Hickie I. B. Kemp A. H. (2013a). Moderate alcohol intake is related to increased heart rate variability in young adults: implications for health and well-being. *Psychophysiology* 50 1202–1208. 10.1111/psyp.12134 23941125

[B102] Quintana D. S. Heathers J. A. (2014). Considerations in the assessment of heart rate variability in biobehavioral research. *Front. Psychol.* 5:805 10.3389/fpsyg.2014.00805 PMC4106423 25101047

[B103] Quintana D. S. McGregor I. S. Guastella A. J. Malhi G. S. Kemp A. H. (2013b). A meta-analysis on the impact of alcohol dependence on short-term resting-state heart rate variability: implications for cardiovascular risk. *Alcohol. Clin. Exp. Res.* 37(Suppl. 1), E23–E29. 10.1111/j.1530-0277.2012.01913.x 22834996

[B104] Raj S. R. Roach D. E. Koshman M. L. Sheldon R. S. (2004). Activity-responsive pacing produces long-term heart rate variability. *J. Cardiovasc. Electrophysiol.* 15 179–183. 10.1111/j.1540-8167.2004.03342.x 15028048

[B105] Rebelo A. C. Tamburus N. Salviati M. Celante V. Takahashi A. de Sa M. (2011). Influence of third-generation oral contraceptives on the complexity analysis and symbolic dynamics of heart rate variability. *Eur. J. Contracept. Reprod. Health Care* 16 289–297. 10.3109/13625187.2011.591217 21774565

[B106] Riniolo T. Porges S. W. (1997). Inferential and descriptive influences on measures of respiratory sinus arrhythmia: sampling rate, R-wave trigger accuracy, and variance estimates. *Psychophysiology* 34 613–621. 9299916 10.1111/j.1469-8986.1997.tb01748.x

[B107] Roach D. Sheldon A. Wilson W. Sheldon R. (1998). Temporally localized contributions to measures of large-scale heart rate variability. *Am. J. Physiol.* 274(5 Pt 2), H1465–H1471. 9612351 10.1152/ajpheart.1998.274.5.H1465

[B108] Roach D. Wilson W. Ritchie D. Sheldon R. (2004). Dissection of long-range heart rate variability: controlled induction of prognostic measures by activity in the laboratory. *J. Am. Coll. Cardiol.* 43 2271–2277. 10.1016/j.jacc.2004.01.050 15193692

[B109] Rodriguez-Linares L. Lado M. J. Vila X. A. Mendez A. J. Cuesta P. (2014). gHRV: heart rate variability analysis made easy. *Comput. Methods Programs Biomed.* 116 26–38. 10.1016/j.cmpb.2014.04.007 24854108

[B110] Rottenberg J. Salomon K. Gross J. J. Gotlib I. H. (2005). Vagal withdrawal to a sad film predicts subsequent recovery from depression. *Psychophysiology* 42 277–281. 10.1111/j.1469-8986.2005.00289.x 15943681

[B111] Saboul D. Pialoux V. Hautier C. (2014). The breathing effect of the LF/HF ratio in the heart rate variability measurements of athletes. *Eur. J. Sport Sci.* 14(Suppl. 1), S282–S288. 10.1080/17461391.2012.691116 24444219

[B112] Salahuddin L. Cho J. Jeong M. G. Kim D. (2007). Ultra short term analysis of heart rate variability for monitoring mental stress in mobile settings. *Conf. Proc. IEEE Eng. Med. Biol. Soc.* 2007 4656–4659. 10.1109/iembs.2007.4353378 18003044

[B113] Sassi R. Cerutti S. Lombardi F. Malik M. Huikuri H. V. Peng C. (2015). Advances in heart rate variability signal analysis: joint position statement by the e-Cardiology ESC Working Group and the European Heart Rhythm Association co-endorsed by the Asia Pacific Heart Rhythm Society. *Europace* 17 1341–1353. 10.1093/europace/euv015 26177817

[B114] Saus E. R. Johnsen B. H. Eid J. Riisem P. K. Andersen R. Thayer J. F. (2006). The effect of brief situational awareness training in a police shooting simulator: an experimental study. *Mil. Psychol.* 18 S3–S21.

[B115] Saus E. R. Johnsen B. H. Eid J. Thayer J. F. (2012). Who benefits from simulator training: personality and heart rate variability in relation to situation awareness during navigation training. *Comput. Hum. Behav.* 28 1262–1268. 10.1016/j.chb.2012.02.009

[B116] Schafer A. Vagedes J. (2013). How accurate is pulse rate variability as an estimate of heart rate variability? A review on studies comparing photoplethysmographic technology with an electrocardiogram. *Int. J. Cardiol.* 166 15–29. 10.1016/j.ijcard.2012.03.119 22809539

[B117] Schroeder E. B. Liao D. Chambless L. E. Prineas R. J. Evans G. W. Heiss G. (2003). Hypertension, blood pressure, and heart rate variability: the atherosclerosis risk in communities (ARIC) study. *Hypertension* 42 1106–1111. 10.1161/01.HYP.0000100444.71069.73 14581296

[B118] Schulz S. M. Ayala E. Dahme B. Ritz T. (2009). A MATLAB toolbox for correcting within-individual effects of respiration rate and tidal volume on respiratory sinus arrhythmia during variable breathing. *Behav. Res. Methods* 41 1121–1126. 10.3758/BRM.41.4.1121 19897819

[B119] Schwerdtfeger A. Derakshan N. (2010). The time line of threat processing and vagal withdrawal in response to a self-threatening stressor in cognitive avoidant copers: evidence for vigilance-avoidance theory. *Psychophysiology* 47 786–795. 10.1111/j.1469-8986.2010.00965.x 20136733

[B120] Shaffer F. Combatalade D. C. (2013). Don’t add or miss a beat: a guide to cleaner heart rate variability recordings. *Biofeedback* 41 121–130. 10.5298/1081-5937-41.3.04

[B121] Shaffer F. McCraty R. Zerr C. L. (2014). A healthy heart is not a metronome: an integrative review of the heart’s anatomy and heart rate variability. *Front. Psychol.* 5:1040 10.3389/fpsyg.2014.01040 PMC4179748 25324790

[B122] Sjoberg N. Saint D. A. (2011). A single 4 mg dose of nicotine decreases heart rate variability in healthy nonsmokers: implications for smoking cessation programs. *Nicotine Tob. Res.* 13 369–372. 10.1093/ntr/ntr004 21350044

[B123] Smith A. L. Owen H. Reynolds K. J. (2013a). Heart rate variability indices for very short-term (30 beat) analysis. Part 1: survey and toolbox. *J. Clin. Monit. Comput.* 27 569–576. 10.1007/s10877-013-9471-4 23674071

[B124] Smith A. L. Owen H. Reynolds K. J. (2013b). Heart rate variability indices for very short-term (30 beat) analysis. Part 2: validation. *J. Clin. Monit. Comput.* 27 577–585. 10.1007/s10877-013-9473-2 23681923

[B125] Soares-Miranda L. Sattelmair J. Chaves P. Duncan G. E. Siscovick D. S. Stein P. K. (2014). Physical activity and heart rate variability in older adults: the cardiovascular health study. *Circulation* 129 2100–2110. 10.1161/CIRCULATIONAHA.113.005361 24799513 PMC4038662

[B126] Stanley J. Peake J. M. Buchheit M. (2013a). Cardiac parasympathetic reactivation following exercise: implications for training prescription. *Sports Med.* 43 1259–1277. 10.1007/s40279-013-0083-4 23912805

[B127] Stanley J. Peake J. M. Buchheit M. (2013b). Consecutive days of cold water immersion: effects on cycling performance and heart rate variability. *Eur. J. Appl. Physiol.* 113 371–384. 10.1007/s00421-012-2445-2 22752345

[B128] Stein P. K. Domitrovich P. P. Hui N. Rautaharju P. Gottdiener J. (2005). Sometimes higher heart rate variability is not better heart rate variability: results of graphical and nonlinear analyses. *J. Cardiovasc. Electrophysiol.* 16 954–959. 10.1111/j.1540-8167.2005.40788.x 16174015

[B129] Stein P. K. Pu Y. (2012). Heart rate variability, sleep and sleep disorders. *Sleep Med. Rev.* 16 47–66. 10.1016/j.smrv.2011.02.005 21658979

[B130] Swan M. (2013). The quantified self: fundamental disruption in big data science and biological discovery. *Big Data* 1 85–99. 10.1089/big.2012.0002 27442063

[B131] Tak L. M. Riese H. de Bock G. H. Manoharan A. Kok I. C. Rosmalen J. G. M. (2009). As good as it gets? A meta-analysis and systematic review of methodological quality of heart rate variability studies in functional somatic disorders. *Biol. Psychol.* 82 101–110. 10.1016/j.biopsycho.2009.05.002 19463887

[B132] Tarvainen M. P. Niskanen J. P. (2012). *Kubios HRV Version 2.1 – User’s Guide.* Kuopio: University of Eastern Finland.

[B133] Tarvainen M. P. Niskanen J. P. Lipponen J. A. Ranta-Aho P. O. Karjalainen P. A. (2014). Kubios HRV–heart rate variability analysis software. *Comput. Methods Programs* 113 210–220. 10.1016/j.cmpb.2013.07.024 24054542

[B134] Thayer J. F. Ahs F. Fredrikson M. Sollers J. J. Wager T. D. (2012). A meta-analysis of heart rate variability and neuroimaging studies: implications for heart rate variability as a marker of stress and health. *Neurosci. Biobehav. Rev.* 36 747–756. 10.1016/j.neubiorev.2011.11.009 22178086

[B135] Thayer J. F. Hansen A. L. Saus-Rose E. Johnsen B. H. (2009). Heart rate variability, prefrontal neural function, and cognitive performance: the neurovisceral integration perspective on self-regulation, adaptation, and health. *Ann. Behav. Med.* 37 141–153. 10.1007/s12160-009-9101-z 19424767

[B136] Thayer J. F. Lane R. D. (2000). A model of neurovisceral integration in emotion regulation and dysregulation. *J. Affect. Disord.* 61 201–216. 10.1016/S0165-0327(00)00338-4 11163422

[B137] Thayer J. F. Loerbroks A. Sternberg E. M. (2011). Inflammation and cardiorespiratory control: the role of the vagus nerve. *Respir. Physiol. Neurobiol.* 178 387–394. 10.1016/j.resp.2011.05.016 21642019

[B138] Thayer J. F. Sollers J. J. III Ruiz-Padial E. Vila J. (2002). Estimating respiratory frequency from autoregressive spectral analysis of heart period. *IEEE Eng. Med. Biol. Mag.* 21 41–45. 10.1109/memb.2002.1032638 12222116

[B139] Thayer J. F. Wang X. Snieder H. (2006). Ethnic differences in heart rate variability: Does ultralow-frequency heart rate variability really measure autonomic tone? *Am. Heart J.* 152:e27 10.1016/j.ahj.2006.05.027 16923401

[B140] Umetani K. Singer D. H. McCraty R. Atkinson M. (1998). Twenty-four hour time domain heart rate variability and heart rate: relations to age and gender over nine decades. *J. Am. Coll. Cardiol.* 31 593–601. 9502641 10.1016/s0735-1097(97)00554-8

[B141] van Eekelen A. P. Houtveen J. H. Kerkhof G. A. (2004). Circadian variation in cardiac autonomic activity: reactivity measurements to different types of stressors. *Chronobiol. Int.* 21 107–129. 15129827 10.1081/cbi-120027983

[B142] Verkuil B. Brosschot J. F. Tollenaar M. S. Lane R. D. Thayer J. F. (2016). Prolonged non-metabolic heart rate variability reduction as a physiological marker of psychological stress in daily life. *Ann. Behav. Med.* 50 704–714. 10.1007/s12160-016-9795-7 27150960 PMC5054058

[B143] Villarejo M. V. Zapirain B. G. Zorrilla A. M. (2013). Algorithms based on CWT and classifiers to control cardiac alterations and stress using an ECG and a SCR. *Sensors (Basel)* 13 6141–6170. 10.3390/s130506141 23666135 PMC3690048

[B144] Weippert M. Kumar M. Kreuzfeld S. Arndt D. Rieger A. Stoll R. (2010). Comparison of three mobile devices for measuring R-R intervals and heart rate variability: polar S810i, Suunto t6 and an ambulatory ECG system. *Eur. J. Appl. Physiol.* 109 779–786. 10.1007/s00421-010-1415-9 20225081

[B145] Wilhelm F. H. Grossman P. Coyle M. A. (2004). Improving estimation of cardiac vagal tone during spontaneous breathing using a paced breathing calibration. *Biomed. Sci. Instrum.* 40 317–324. 15133978

[B146] Yi S. H. Lee K. Shin D. G. Kim J. S. Kim H. C. (2013). Differential association of adiposity measures with heart rate variability measures in Koreans. *Yonsei Med. J.* 54 55–61. 10.3349/ymj.2013.54.1.55 23225799 PMC3521274

[B147] Young F. L. Leicht A. S. (2011). Short-term stability of resting heart rate variability: influence of position and gender. *Appl. Physiol. Nutr. Metab.* 36 210–218. 10.1139/h10-103 21609282

[B148] Zimmermann-Viehoff F. Thayer J. F. Koenig J. Herrmann C. Weber C. S. Deter H. C. (2015). Short-term effects of espresso coffee on heart rate variability and blood pressure in habitual and non-habitual coffee consumers - A randomized crossover study. *Nutr. Neurosci.* 19 169–175. 10.1179/1476830515Y.0000000018 25850440

