# Supplementary material for: Heart Rate Variability and Cardiac Vagal Tone in Psychophysiological Research – Recommendations for Experiment Planning, Data Analysis, and Data Reporting
Source: Front Psychol. 2017 Feb 20;8:213. doi: 10.3389/fpsyg.2017.00213 (PMC5316555; doi:10.3389/fpsyg.2017.00213)
Supplement: Supplementary file 1 [file Data_Sheet_1.DOCX]

Demographic Questionnaire - HRV Psychophysiological Experiment

Please answer the questions honestly. Your answers will remain anonymous.

**Gender:** Male / Female **Age:** ____________

|  | **YES**  **NO** |
| --- | --- |
| 1. Have you rushed in order to arrive on time for this experiment? | ❒ ❒ |
| 1. Have you taken part in any intensive physical activity in the past 24 hours? If yes please describe activity type and length. | ❒ ❒ |
| 1. When was the last time you exercised? |  |
| 1. Have you eaten in the past two hours? | ❒ ❒ |
| 1. Have you consumed any caffeine/theine-containing beverages in the past two hours? | ❒ ❒ |
| 1. Have you consumed any alcoholic beverages in the past 24 hours? | ❒ ❒ |
| 1. Do you usually smoke?   If yes, please report the number of cigarettes you smoke on a daily basis. | ❒ ❒  _________________________________________ |
| 1. Have you smoked in the past two hours? | ❒ ❒ |
| 1. Do you currently take any medication?   If yes, please write down the name of the medication/s. | ❒ ❒  _________________________________________ |
| 1. For female participants, are you taking a form of oral contraceptive? | ❒ ❒ |
| 1. Do you have any known blood pressure conditions? | ❒ ❒ |
| 1. Did you follow your usual sleep routine last night? | ❒ ❒ |
| 1. When did you get up this morning? |  |
| 1. When did you go to sleep last night? |  |
| 1. Do you suffer from any mental disorders, for example severe depression or anxiety disorder? | ❒ ❒ |
| 1. Do you have any chronic heart issues or respiratory conditions? | ❒ ❒ |
| 1. Do you need to use the bathroom? | ❒ ❒ |

Height: ____________ Weight: ____________

Hips measurement: ____________ Waist measurement: ____________
