# Supplementary material for: Heart Rate Variability and Cardiac Vagal Tone in Psychophysiological Research – Recommendations for Experiment Planning, Data Analysis, and Data Reporting
Source: Front Psychol. 2017 Feb 20;8:213. doi: 10.3389/fpsyg.2017.00213 (PMC5316555; doi:10.3389/fpsyg.2017.00213)
Supplement: Supplementary file 2 [file Data_Sheet_2.DOCX]

**Experimental Protocol: Working memory, reinvestment, and vagal tone**

**Experimental condition: *No-pressure condition***

Date/Time: ______________ Participant ID: ______________

Randomization code: ______________

| **EXPERIMENT PROTOCOL** | |
| --- | --- |
| **Researcher instructions** | **Done?** |
| **AT LEAST ONE DAY BEFORE THE EXPERIMENT** | |
| Send instructions via email regarding experiment organization and which rules to observe before the experiment (e.g. no food two hours before). |  |
| **EXPERIMENT DAY: BEFORE PARTICIPANT ARRIVES** | |
| Start laptop |  |
| Prepare non-moving chair for participant |  |
| Get tissue ready for the participant to wipe away the gel from the electrodes |  |
| Put a ”do not disturb” sign on the lab door |  |
| Check randomization order on randomization list |  |
| Prepare documents related to the experiment:   - Reinvestment questionnaires - Visual analogue scales |  |
| Get ECG-device (eMotion HRV) ready |  |
| Get external clock ready (to write down event times – *the clock needs to display hours:minutes:seconds*) |  |
| 1. Plug in eMotion HRV device to the computer 2. Start the eMotion Manager software 3. Check eMotion HRV device battery, if necessary wait for charging until it reaches at least 50% 4. Synchronize computer time with eMotion HRV device time by clicking synchronize 5. Synchronize external clock time with computer time |  |
| Prepare two ECG electrodes |  |
| Plug in headphones and check functioning |  |
| Prepare playlist with experimental instructions as audio files in Windows Media Player |  |
| Check the volume of the computer, windows media player and headphones |  |
| **WELCOME PARTICIPANT TO EXPERIMENT** | |
| Explain procedure/ give information sheet/ any questions? |  |
| Sign the informed consent form |  |
| Ask the participant to turn off their mobile phone |  |
| Fill out demographic questionnaire |  |
| Fill out reinvestment questionnaires |  |
| **ECG MEASUREMENT PREPERATION** | |
| Attach the first electrode (in the right infraclavicular fossa, just below the right clavicle) |  |
| Attach the second electrode (on the left side of the chest, below the pectoral muscle in the left anterior axillary line) |  |
| Plug in upper clip of eMotion HRV device |  |
| Plug in lower clip of eMotion HRV device |  |
| Prompt: *“This is the last chance to go to the bathroom for the next hour”* |  |
| Turn on eMotion HRV device (press and hold the main button for 2 seconds) and write down starting time | **TIME: _____:_____:_____** |
| Check whether the green light is blinking on the eMotion HRV device (means eMotion HRV device is on, and corresponds to heart rate recording) |  |
| **PARTICIPANT TAKES SEAT IN FRONT OF THE COMPUTER** | |
| Prompt: *“You can put the headphones on and we will start”* |  |
| Play baselines instructions in Windows Media Player |  |
| **BASELINE MEASUREMENT (5 min)** | |
| Baseline start | **TIME: _____:_____:_____** |
| Baseline end | **TIME: _____:_____:_____** |
| Hand out questionnaire (VAS): stress: _______ |  |
| Play task instructions in Windows Media Player |  |
| Start AOSPAN Task with Inquisit Software |  |
| START Practice AOSPAN Task | **TIME: _____:_____:_____** |
| START Real AOSPAN Task | **TIME: _____:_____:_____** |
| END Real AOSPAN Task | **TIME: _____:_____:_____** |
| **POST WORKING-MEMORY TASK - RECOVERY MEASUREMENT (5min)** | |
| Hand out questionnaire (VAS): stress: _______ |  |
| Play recovery instructions in Windows Media Player |  |
| Recovery start | **TIME: _____:_____:_____** |
| Recovery end | **TIME: _____:_____:_____** |
| Turn off eMotion HRV device (press and hold main button for 5s) |  |
| Disconnect eMotion HRV device from electrodes |  |
| Remove electrodes and provide participant with tissue |  |
| Thank and debrief participant |  |
| **STEPS AFTER THE EXPERIMENT** | |
| Plug eMotion HRV device to computer |  |
| Open eMotion HRV software  Save .sdf Data and export data files to separate folder  (Data name: Participant ID_Condition_HRV time) |  |
| Backup copy of data files on the University server |  |
| Put eMotion HRV device, headset, clock, electrodes, and laptop back in lab cupboard |  |
| Tidy up the lab |  |
| Take off “Do not disturb” sign from lab door |  |
| **END** | |

*Note:* The right column serves as indicating a “Check” when the action has been performed, to ensure nothing is forgotten. HRV: Heart Rate Variability; VAS: Visual Analogue Scale
