# Supplementary material for: Heart Rate Variability and Cardiac Vagal Tone in Psychophysiological Research – Recommendations for Experiment Planning, Data Analysis, and Data Reporting
Source: Front Psychol. 2017 Feb 20;8:213. doi: 10.3389/fpsyg.2017.00213 (PMC5316555; doi:10.3389/fpsyg.2017.00213)
Supplement: Supplementary file 3 [file Data_Sheet_3.DOCX]

Supplementary material

*Figure 1.* Comparison manual vs. automatic artefact correction with Kubios


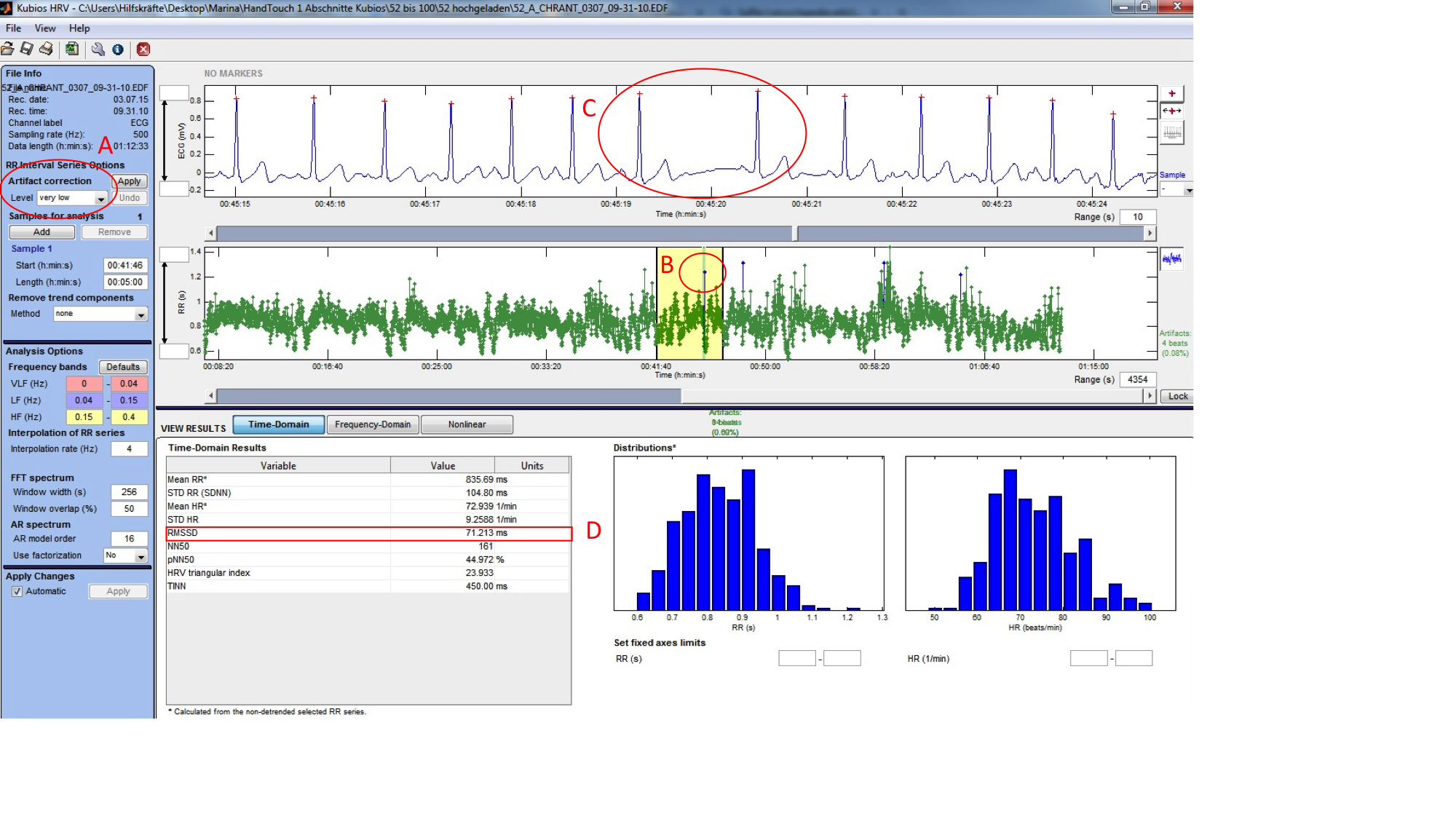


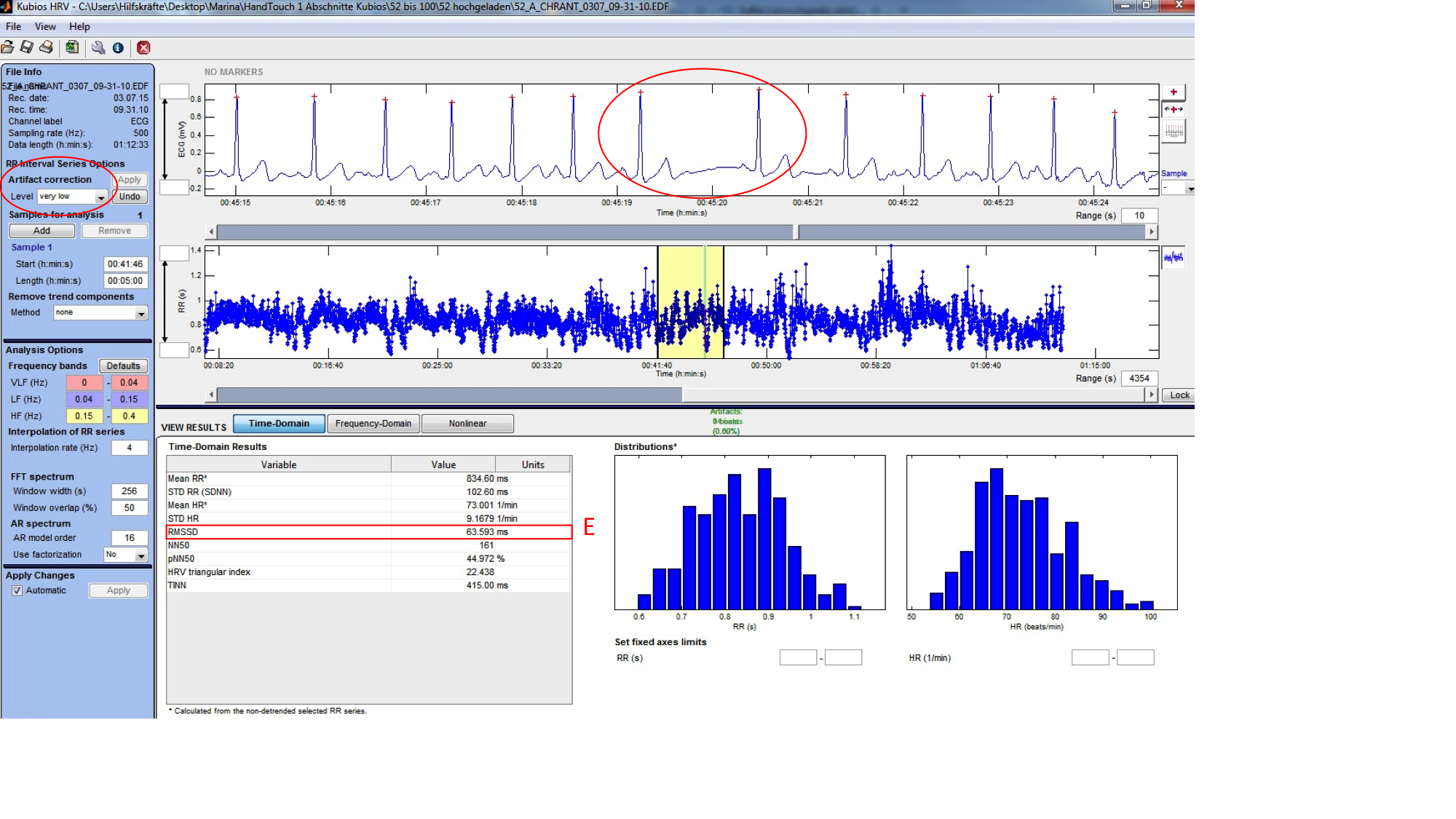
This figure displays the issues that might be raised when artefacts are corrected automatically by a software like Kubios, in case only the RR signal was recorded and not the electrocardiogram (ECG). Here, we see that when using the “very low” automatic artefact correction filter (A), Kubios identifies as an artefact (B) as something that is actually not an artefact as displayed in the ECG signal (C). In case we would apply the automatic correction, the RMSSD value (D) that is originally of 71.213 ms would drop to 63.593 ms (E), which represents a change of 11%.
